# Double dative bond between divalent carbon(0) and uranium

**DOI:** 10.1038/s41467-018-07377-6

**Published:** 2018-11-27

**Authors:** Wei Su, Sudip Pan, Xiong Sun, Shuao Wang, Lili Zhao, Gernot Frenking, Congqing Zhu

**Affiliations:** 1 State Key Laboratory of Coordination Chemistry, Jiangsu Key Laboratory of Advanced Organic Materials, School of Chemistry and Chemical Engineering, Nanjing University, 210023 Nanjing, China; 2 Institute of Advanced Synthesis, School of Chemistry and Molecular Engineering, Jiangsu National Synergetic Innovation Center for Advanced Materials, Nanjing Tech University, 211816 Nanjing, China; 3 State Key Laboratory of Radiation Medicine and Protection, School for Radiological and interdisciplinary Sciences (RAD-X) and Collaborative Innovation Center of Radiation Medicine of Jiangsu Higher Education Institutions, Soochow University, 199 Ren’ai Road, 215123 Suzhou, China; 4 Fachbereich Chemie, Philipps-Universität Marburg, Hans-Meerwein-Straße 4, 35032 Marburg, Germany; 5 Donostia International Physics Center (DIPC), P.K. 1072, 20080 Donostia, Euskadi, Spain

## Abstract

Dative bonds between *p*- and *d*-block atoms are common but species containing a double dative bond, which donate two-electron pairs to the same acceptor, are far less common. The synthesis of complexes between UCl_4_ and carbodiphosphoranes (CDP), which formally possess double dative bonds Cl_4_U⇇CDP, is reported in this paper. Single-crystal X-ray diffraction shows that the uranium−carbon distances are in the range of bond lengths for uranium−carbon double bonds. A bonding analysis suggests that the molecules are uranium−carbone complexes featuring divalent carbon(0) ligands rather than uranium−carbene species. The complexes represent rare examples with a double dative bond in *f*-block chemistry. Our study not only introduces the concept of double dative bonds between carbones and *f*-block elements but also opens an avenue for the construction of other complexes with double dative bonds, thus providing new opportunities for the applications o*f f*-block compounds.

## Abstract (web summary)

While chemical bonding between carbon and the *d*- and *p*-block elements is relatively well-studied, that between carbon and the *f*-block elements remains comparatively poorly understood. Here, the authors synthesize a series of uranium−carbone complexes in which carbon forms an unprecedented double dative bond to uranium.

## Introduction

Understanding the nature of chemical bonding is of great importance, especially for the bonding of carbon with other elements^1–4^. Usually, carbon uses all of its four valence electrons to form stable tetravalent carbon(IV) species. Since the first stable singlet carbene at room temperature was isolated by Bertrand and coworkers^3^, divalent carbon(II) chemistry, such as N-heterocyclic carbene (NHC)^6–9^ and cyclic (alkyl)(amino)carbenes^10–13^, has flourished. Carbenes, CR_2_, which contain one lone electron pair, can coordinate to numerous main group atoms and *d*-block or even *f*-block elements forming stable species via a single dative bond. If all four carbon valence electrons are retained as two-electron pairs, divalent carbon(0) compounds CL_2_ (“carbones”) are formed^14–25^. Thus, the fundamental difference between a carbene CR_2_ and a carbone CL_2_ is the number of electron lone-pairs that may serve as donors: carbenes are two-electron (single lone-pair) donors while carbones are four-electron (double lone-pair) donors. Although the first divalent carbon(0) species^9^, carbodiphosphorane (CDP), was reported already in 1961, the actual bonding situation was not fully understood until a theoretical study appeared in 2006 by Frenking and coworkers^27–29^. They showed that CDP can be considered as two phosphine ligands coordinated to a carbon atom in the excited ^1^D state with two lone electron pairs, which remain available for both σ- and π-donation simultaneously^13^. There are numerous complexes in which carbones bind to two acceptors^31–33^. In contrast, examples containing a double dative bond, which donates two lone electron pairs to the same acceptor are rare^34–37^. A comparative experimental work showed that carbenes and carbones exhibit distinctively different complexation behavior due to the varying number of lone-pair orbitals^32^. The chemistry of divalent carbon(0) has been focused mainly on main group compounds and transition metal element complexes. The bonding motif between divalent carbon(0) and *f*-block elements has received less attention. Since uranium plays fascinating roles in energy and catalysis research^38–43^, it is important to isolate species containing a new uranium−carbon bond, which could provide a deeper understanding of the nature of chemical bonding between main group elements and *f*-block metals.

In this paper, we report a set of complexes that possess a double dative bond between carbon and uranium. The complexes were obtained by the reaction of different CDPs with uranium tetrachloride (UCl_4_). Single-crystal X-ray diffraction reveals that the molecules possess rather short uranium−carbon bonds. Density functional theory (DFT) calculations confirm the double dative bond feature between carbon and uranium. The results of this study could be useful for the design of other compounds containing *f*-block atoms that exhibit double dative bonds between the metal and carbone ligands, and could provide new opportunities for the applications of *f*-block elements in catalysis or in the activation of small molecules.

## Results

### Synthesis and characterization

**Fig. 1:**
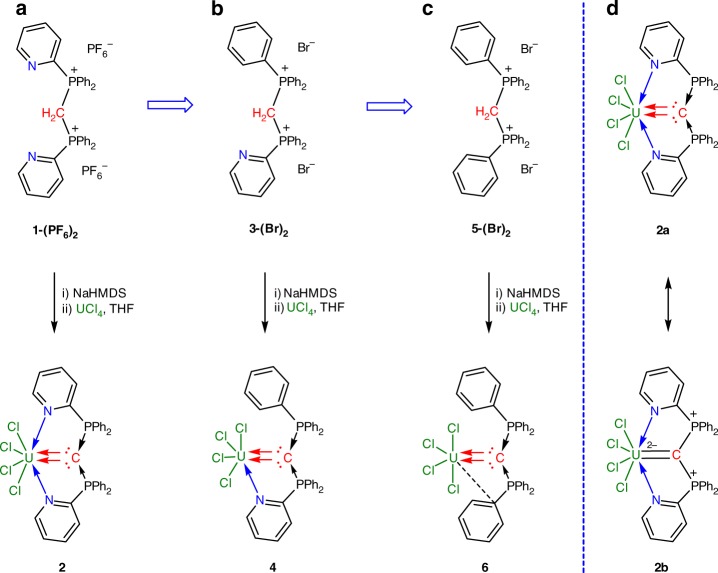
Synthesis of CDP-UCl_4_ adducts. Synthesis of double dative bond adducts **2** (**a**), **4** (**b**), and **6** (**c**) employ tridentate, bidentate, and monodentate CDP precursors **1-(PF**_**6**_**)**_**2**_, **3-(Br)**_**2**_, and **5-(Br)**_**2**_, respectively. **d** Two major resonance structures of complex **2** with a double dative bond (**2a**) and with an electron-sharing U=C double bond (**2b**)

We first designed and prepared a new tridentate CDP precursor, **1-(PF**_**6**_**)**_**2**_, with two 2-(pyridyl)diphenylphosphine units (see Supplementary Methods). The corresponding CDP-UCl_4_ adduct (**2**) was readily synthesized by the deprotonation of compound **1-(PF**_**6**_**)**_**2**_ with NaHMDS and subsequent reaction in situ of the resulting CDP with UCl_4_ (Fig. 1a). Complex **2** was isolated in 66% yield as yellow-green crystals after recrystallization at ‒35 °C overnight. The crystalline form of **2** shows low solubility in most organic solvents and easily decomposes to the free ligand. Consequently, the ^31^P nuclear magnetic resonance (NMR) spectrum shows two equivalent phosphine signals at ‒215.87 ppm for complex **2** along with the signal for **1** (Supplementary Fig. 1). The ^1^H NMR spectrum of complex **2** exhibits a broad range of peaks from +14.19 to −41.54 ppm (Supplementary Fig. 2).

**Fig. 2:**
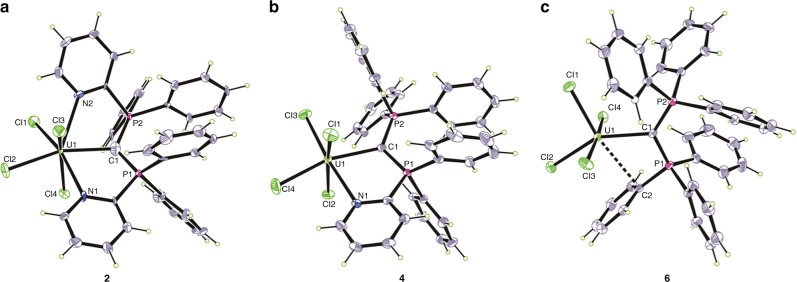
Molecular structures of CDP-UCl_4_ adducts **2**, **4**, and **6**. **a**–**c** Solid-state structures of **2** (**a**), **4** (**b**), and **6** (**c**) by X-ray crystallography with 50% probability ellipsoids. Solvent molecules are omitted for clarity. Selected experimental [calculated] bond distances (Å) and angles (deg) for **2**: U1–C1 2.471(7) [2.425], U1–N1 2.625(5) [2.612], U1–N2 2.582(6) [2.577], U1–Cl1 2.649(2) [2.612], U1–Cl2 2.645(2) [2.577], U1–Cl3 2.644(2) [2.663], U1–Cl4 2.655(2) [2.651], C1–P1 1.701(7) [1.680], C1–P2 1.690(6) [1.684], P1-C1-P2 121.3(4) [123.0]. For **4**: U1–C1 2.461(5) [2.461], U1–N1 2.537(4) [2.530], U1–Cl1 2.598(2) [2.617], U1–Cl2 2.570(2) [2.615], U1–Cl3 2.592(2) [2.566], U1–Cl4 2.622(2) [2.570], C1–P1 1.711(5) [1.692], C1–P2 1.699(5) [1.711], P1-C1-P2 120.6(3) [117.7]. For **6**: U1–C1 2.411(3) [2.436], U1–Cl1 2.550(1) [2.544], U1–Cl2 2.604(1) [2.554], U1–Cl3 2.594(1) [2.603], U1–Cl4 2.634(1) [2.609], C1–P1 1.705(3) [1.699], C1–P2 1.719(3) [1.710], P1-C1-P2 125.05(16) [121.9]. Uranium, yellow-green; phosphorus, violet red; nitrogen, blue; chlorine, green; carbon, gray

The solid-state structure of **2** was confirmed by an analysis of its X-ray diffraction pattern (Fig. 2a). The uranium atom adopts a twisted pentagonal bipyramidal geometry in which both pyridyl ligands are coordinated to the uranium center forming two five-membered rings. The average U−N and U−Cl bond lengths are 2.604(5) Å and 2.648(2) Å, respectively. The bond length of U1−C1 was found to be 2.471(7) Å, which is obviously shorter than the U−C bond distances found in the adducts of NHC with uranium (2.573 Å−2.788 Å) (based on a search of the Cambridge Structural Database, CSD version 5.39 (updates 2017)). This U−C bond length in complex **2** (2.471(7) Å) is in the range of U(IV)=C double bond distances (2.310 Å−2.578 Å) in complexes with bis(iminophosphorano)methandiide as ligands (based on a search of the Cambridge Structural Database, CSD version 5.39 (updates 2017)). An alternative description of **2** with a double bond between uranium and carbon is provided by the resonance structure **2b** (Fig. 1d). This depicts a carbon(IV) atom with an electron-sharing U=C double bond in which uranium has a formal charge of −2. Analysis of the bonding suggests that **2a** is the dominant resonance form contributing to the bonding situation.

The short U−C bond in complex **2** prompted us to appraise uranium−carbon multiple bond chemistry^44–46^. The first uranium carbene was reported in 1981, and was stabilized by a phosphorus substituent^29^. Subsequently, various species containing a uranium carbon double bond with one or two phosphorus substituents on the carbene carbon were reported^48–63^. Interestingly, most of the reported uranium carbenes utilize tridentate chelating methanediide ligands^47–64]^. Indeed, some of the uranium carbene species in the literature might be considered to contain non-negligible carbone character in their bonding^49,56,54^. In addition, species containing a U−C double bond, triple bond, or even quadruple bond were identified by matrix infrared spectroscopy and/or relativistic density functional calculations^64–69^. Very recently, a diuranium carbide cluster (U=C=U) was also stabilized inside a C_80_ fullerene cage^50^.

In order to investigate the effect of CDP ligands on the bonding between divalent carbon(0) and uranium, analogous bidentate and monodentate CDP precursors **3-(Br)**_**2**_ and **5-(Br)**_**2**_ were synthesized. As shown in Fig. 1b, c, treatment of UCl_4_ with the CDP ligands, generated by the in situ deprotonation of **3-(Br)**_**2**_ and **5-(Br)**_**2**_ with NaHMDS in THF, resulted in the formation of CDP-UCl_4_ adducts **4** and **6** in 52% and 59% crystalline yields, respectively, after recrystallization at −35 °C. Both crystals of complexes **4** and **6** totally decomposed after re-dissolved in common solvents. Fortunately, the ^31^P NMR spectrum of complex **4** was recorded from the in situ reaction solutions. The signals of two nonequivalent phosphorus centers in this species were observed at ‒125.51 and ‒155.13 ppm (Supplementary Fig. 3). Complex **6** represents an unusual non-chelated species with a double dative bond between carbon and an *f*-block element compared with reported work^47,48,67^, but its stability is significantly lower than that of chelating complexes **2** and **4**.

The crystal structure of complex **4** (Fig. 2b) reveals a U1−C1 bond distance of 2.461(5) Å, which compares well with that found in complex **2**. The bond distances of U1−N1 (2.537(4) Å) and average U−Cl (2.595(2) Å) in **4** were slightly shorter than those observed in complex **2**. The pyridyl unit coordinates with uranium center to form a five-membered ring, generating a distorted octahedral configuration. An X-ray study of **6** showed the uranium to have a distorted octahedral structure, concerning a weak interaction between the uranium center and a phenyl carbon atom, with a U1−C1 bond length of 2.411(3) Å (Fig. 2c). The bond lengths of U1−C1 in all these complexes are obviously shorter than the U−C distances (2.573 Å−2.788 Å) typically observed in NHC-uranium adducts. These data suggest that the bond between the divalent carbon(0) of CDP and the uranium is a multiple bond.

**Fig. 3:**
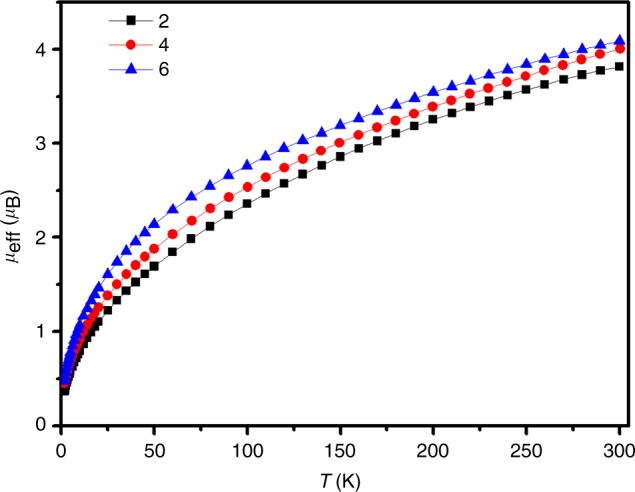
Magnetic characterization data. Variable-temperature SQUID magnetization data of complex **2** (black square), complex **4** (red circle), and complex **6** (blue triangle) at 0.1 T. Lines are a guide to the eye only

The recording of reliable absorption spectra of these complexes is prevented by their poor stability in solution. Fourier-transform infrared (FT-IR) spectra demonstrated bands at 690 cm^−1^ for **2**, 687 cm^−1^ for **4**, and 686 cm^−1^ for **6**, respectively. These data match excellently with those computed for U–C stretching (674 cm^−1^ for **2**, 673 cm^−1^ for **4**, and 671 cm^−1^ for **6**, Supplementary Table 3), suggesting the intrinsic double dative bond between uranium center and carbone carbon atom. Furthermore, variable-temperature magnetic measurements were used to characterize these species in the solid state. The magnetic moment of **2** at 300 K is approximately 3.81 *μ*_B_, which is slightly higher than the expected value (3.58 *μ*_B_) for the ^3^H_4_ ground state of *f*^2^ uranium(IV). Upon cooling, the magnetic moments decrease smoothly to 0.37 *μ*_B_ at 1.8 K and tending to zero (Fig. 3 and Supplementary Figs. 4-6). The magnetic moments and trends of **4** and **6** are similar to those of **2**, which are consistent with uranium(IV) centers in those species. The uranium(IV) centers in these complexes were further confirmed by the field-dependent magnetization data collected at 1.8 K (Supplementary Figs. 7–9).

### Theoretical studies

DFT calculations were performed to elucidate the bonding situation in **2**, **4**, and **6**. The geometries of these species were optimized at the BP86-D3(BJ)/def2-TZVPP/Stuttgart RSC ECP level using scalar-relativistic effective core potentials for uranium (see Supplementary Methods). Calculations at various spin states suggest that **2**, **4**, and **6** have an electronic triplet ground state. Calculated structures at the electronic singlet and quintet state are much higher in energy (Supplementary Fig. 10). The calculated bond lengths and angles of the three complexes are in good agreement with the experimental data (Fig. 2). The differences between theory and experiment are within the range of solid-state effects and the accuracy of the methods.

We calculated the bond strength of the uranium−carbon bonds in **2**, **4**, and **6**. The theoretically predicted bond dissociation energies (BDEs) suggest (reactions 1−3) that the introduction of pyridine substituents for phenyl enhances the bond strength, which can be explained with the contribution of the N → U dative bonds to the uranium-CDP bonds. The calculated BDE for **6** (*D*_*e*_ = 70.8 kcal mol^−1^) indicates that the unsupported U⇇C double donor bond is rather strong. Hayton and coworkers^44^ previously reported that the upper limit for the U=C BDE value in the related system is 90 kcal mol^−1^, which is in good agreement with our results. Nonetheless, the values are significantly lower than the BDE value of Ta=C (i.e., 126 kcal mol^−1^) in Ta=CHR(CH_2_R)_3_ (R = SiMe_3_)^52^. This shows the difference in bond strength between bonds involving an actinide and a formal carbone and those with a transition metal and a formal alkylidene.

1 $${{\mathbf{2}}} \to {\mathrm{UCl}}_{\mathrm{4}}\left( {\,}^3{{\mathrm{A}}} \right){\mathrm{ + C}}\left( {{\mathrm{PPh}}_{\mathrm{2}}{\mathrm{Py}}} \right)_{\mathrm{2}}\,{{D}}_{{e}}{\mathrm{ = 91}}{\mathrm{.5}}\,{\mathrm{kcal}}\,{\mathrm{mol}}^{{\mathrm{ - 1}}},$$

2 $${{\mathbf{4}}} \to {\mathrm{UCl}}_{\mathrm{4}}\left( {\,}^3{{\mathrm{A}}} \right){\mathrm{ + C}}\left( {{\mathrm{PPh}}_{\mathrm{2}}{\mathrm{Py}}} \right)\left( {{\mathrm{PPh}}_{\mathrm{3}}} \right) {{D}}_{{e}}{\mathrm{ = 82}}{\mathrm{.8}}\,{\mathrm{kcal}}\,{\mathrm{mol}}^{{\mathrm{ - 1}}},$$

3 $${{\mathbf{6}}} \to {\mathrm{UCl}}_{\mathrm{4}}\left( {\,}^3{{\mathrm{A}}} \right){\mathrm{ + C}}\left( {{\mathrm{PPh}}_{\mathrm{3}}} \right)_{\mathrm{2}} \,{{D}}_{{e}}{\mathrm{ = 70}}{\mathrm{.8}}\,{\mathrm{kcal}}\,{\mathrm{mol}}^{{\mathrm{ - 1}}}.$$

**Fig. 4:**
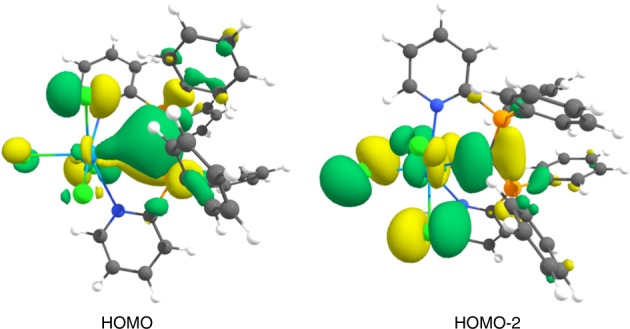
Shape of the HOMO and the HOMO-2 of complex **2**. The isosurface value is 0.03 e Å^−3^

The calculated spin densities in **2**, **4**, and **6** indicate that the unpaired electrons are localized mainly at the uranium atom (Supplementary Fig. 11). This agrees with the shape of the single occupied molecular orbitals (SOMO) and SOMO-1, which are also centered at uranium center (Supplementary Figs. 12-13). Figure 4 shows the highest occupied molecular orbital (HOMO) and HOMO-2 of complex **2**, which may be identified with the π and σ dative bonds of Cl_4_⇇CDP. The related MOs of **4** and **6** are very similar; they are shown in Supplementary Figs. 12–13.

More detailed information about the nature of the Cl_4_U−CDP interactions is available from energy decomposition analysis with natural orbitals for chemical valence (EDA-NOCV) calculations of **2**, **4**, and **6** using the fragments (^3^A) UCl_4_ and singlet CDP with their frozen geometries of the complexes as interacting species. Further details about the method are given in Supplementary Methods. Table 1 shows the numerical results.

**Table 1:** EDA-NOCV results by using the triplet (T) UCl_4_ and singlet (S) CDP fragments

	2 UCl_4_ (T)^+^CDP (S)	4 UCl_4_ (T)^+^CDP (S)	6 UCl_4_ (T)^+^CDP (S)
∆*E*_int_^a^	−133.4	−121.4	−91.9
∆*E*_Pauli_	233.0	187.9	159.2
∆*E*_disp_^b^	−37.6 (10.3%)	−35.4 (11.4%)	−37.5 (14.9%)
∆*E*_elstat_^b^	−186.2 (50.8%)	−154.3 (49.9%)	−125.8 (50.1%)
∆*E*_orb_^b^	−142.6 (38.9%)	−119.5 (38.6%)	−87.9 (35.0%)
∆*E*_orb1_^c^	−39.4 (27.6%)	−37.7 (31.5%)	−27.9 (31.7%)
∆*E*_orb2_^c^	−17.1 (12.0%)	−13.5 (11.3%)	−13.3 (15.1%)
∆*E*_orb3_^c^	−17.1 (12.0%)	−15.2 (12.7%)	−8.6 (9.8%)
∆*E*_orb4_^c^	−14.2 (10.0%)	−11.2 (9.4%)	−6.5 (7.4%)
∆*E*_orb(rest)_^c^	−54.8 (38.5%)	−41.9 (35.0%)	−31.6 (35.9%)

^a^Energy values are given in kcal mol^−1^

^b^The values in parentheses show the contribution towards the total attractive interaction ∆*E*_elstat_ + ∆*E*_orb_ + ∆*E*_disp_

^c^The values in parentheses show the contribution towards the total orbital interaction, ∆*E*_orb_

**Fig. 5:**
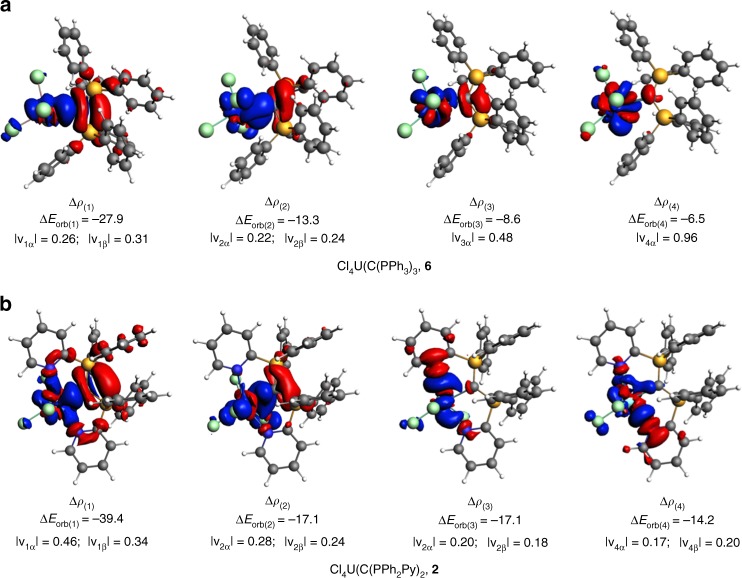
Plot of the deformation densities Δ*ρ* together with the associated interaction energies Δ*E*_orb_. Deformation densities Δ*ρ*_1−4_ of the pairwise orbital interactions in **6** (**a**) and **2** (**b**) between UCl_4_ and the CDP ligand. The energies are in kcal mol^−1^. The charge eigenvalues ν give an estimate of the relative size of the charge migration. The direction of the charge flow is red → blue

The intrinsic interactions energies Δ*E*_int_ exhibit the same trend for the uranium−carbon bond strengths as the BDEs (**2** > **4** > **6**). Inspection of the attractive terms indicates that the dispersion make a non-negligible contribution between 10 and 15% of the total attraction. Furthermore, the uranium−carbon bonds possess a more electrostatic than covalent character as revealed by the relative strength of Δ*E*_elstat_ and Δ*E*_orb_. The most important information comes from the breakdown of the total orbital (covalent) interactions into pairwise orbital contributions Δ*E*_orb(*x*)_. The strongest interactions Δ*E*_orb(1)_ and Δ*E*_orb(2)_ are due to the σ and π components of the double donation Cl_4_U⇇CDP. The nature of the orbital interactions comes to the fore when inspecting the shapes of the associated deformation densities Δ*ρ*_(1)_ and Δ*ρ*_(2)_ in **6**, which are shown in Fig. 5a. There is clearly a charge donation from the σ and π lone-pair electrons on carbon to the uranium atom. The next strongest interactions Δ*E*_orb(3)_ and Δ*E*_orb(4)_ come from the singly occupied orbitals, which involves some further donation from the CDP ligand to uranium. The remaining orbital terms are mainly due to polarization within the fragments.

Figure 5b shows the deformation densities Δ*ρ* of the four strongest pairwise orbital interactions in complex **2**. The stabilization energies Δ*E*_orb(1)_ and Δ*E*_orb(2)_ are again due to the σ and π components of the double donation Cl_4_U⇇CDP. The shape of Δ*ρ*_(3)_ and Δ*ρ*_(4)_ suggest that Δ*E*_orb(3)_ and Δ*E*_orb(4)_ are associated with the additional N → U donation of the pyridine ligands, which have a similar strength as the Cl_4_U ← CDP σ donation. In summary, the EDA-NOCV calculations strongly suggest that the CDP units in **2**, **4**, and **6** serve as double donor ligands toward uranium. A previous study by Liddle and coworkers reported a gallium-uranium complex with a U⇐Ga σ and π bond^53^. They wrote that “π donation by carbene-type fragments may be more widespread than previously recognized”. Here we report an example where the ligand is a carbone CL_2_.

In order to identify the valence atomic orbitals of uranium, which serve as acceptor orbitals for the donor−acceptor interaction with the CDP ligands, we carried out natural bond orbital (NBO) calculations of **2**, **4**, and **6**. Supplementary Fig. 14 shows the shape of two natural bond orbitals that can easily be identified with the σ and π bonds in Cl_4_U⇇CDP. Both bonds are strongly polarized toward the carbon end, which agrees with the findings of Liddle and coworkers in the uranium complexes employing methanediide ligand^54^. Table 2 shows the numerical results of the NBO calculations. Inspection of the hybridization at uranium reveals that the σ and π acceptor orbitals are mainly composed of 5*f* and 6*d* atomic orbitals, with the former being the larger component. Specifically the U−C σ-bond and π-bonds in **2** are polarized with 83% and 91% at the carbon, respectively, which strongly supports the notation of carbon as double donor. The uranium part of the U−C σ-bond has 52% *f* and 39% *d* character. The NBO data for **4** and **6** are very similar to those of complex **2**.

**Table 2:** Calculated NBO compositions of the U−C natural orbitals in the complexes **2**, **4**, and **6**

	σ-bond				π-bond			
	C%	U%	C 2*s*:2*p*	U 7*s*:7*p*:5*f*:6*d*	C%	U%	C 2*s*:2*p*	U 7*s*:7*p*:5 *f*:6*d*
**2**	83	17	33:67	9:0:52:39	91	9	0:100	0:0:54:46
**4**	83	17	34:66	11:0:50:39	91	9	0:100	0:0:53:47
**6**	84	16	33:67	11:0:47:42	89	11	0:100	0:0:55:45

The natural charges and bond orders are provided in Supplementary Table 4 where the charge distribution indicates that 0.64 (**2**), 0.52 (**4**) and 0.50 (**6**) e^−^ are transferred from CDP to UCl_4_ fragment. Therefore, the ligand behavior of CDP is significantly enhanced in the presence of the two pyridyl rings. The C1 center still carries a quite large negative charge (−1.39 to −1.43 e^−^) and this might be due to the significant contribution from its dipolar resonance form and/or the electron density accumulation resulting from an electron transfer from PR_3_ to C1. In principle, both are responsible in varying degrees. The Nalewajski-Mrozek bond order (NMBO)^74–77^, which represents the combined covalent and ionic bond order, has been previously reported to be very well correlated with the U=C bond description. In our study, the NMBO of **2**, **4**, and **6** was computed as 1.30, 1.34, and 1.41, respectively, indicating the double bond character therein.

Finally, the results of quantum theory of atoms in molecules (QTAIM) analysis are provided in Table 3. Note that for covalent bonds, ∇^2^*ρ*(*r*_c_) is usually negative; however, this criterion often fails for the bonds involving heavier elements. This is because ∇^2^*ρ*(*r*_c_) derives from the three curvature values (*λ*_1_, *λ*_2_ and *λ*_3_) where first two terms are negative but *λ*_3_ is positive. For heavier elements, *λ*_3_ term often dominates over the other two terms making the overall ∇^2^*ρ*(*r*_c_) value positive. For these cases, the use of *H*(*r*_c_) is recommended which is usually negative for covalent bonds. In the present cases, although ∇^2^*ρ*(*r*_c_) is positive, the corresponding *H*(*r*_c_) values are negative for U−C1 and U−N bonds. Moreover, the *H*(*r*_c_) value in the former bond is considerably more negative than that in the latter one, indicating stronger covalent interaction in the former case. The weak interaction of the U−C2 (phenyl carbon) bond in **6** is reflected from the positive *H*(*r*_c_) value (Supplementary Fig. 15). We have also computed ellipticity of electron density (*ε*(*r*_c_)) at the BCP of U−C1 bond. In general, for a single bond (σ) and triple bond (σ+2π) which have cylindrical contours of *ρ*, the corresponding *ε*(*r*_c_) value is approximately zero, whereas for a double bond (σ+π), because of the asymmetric distribution of *ρ* in perpendicular to the bond path the *ε*(*r*_c_) value is greater than zero^58^. In the present complexes, the *ε*(*r*_c_) value turned out as 0.09 (**2**), 0.12 (**4**) and 0.11(**6**). Although these values reflect slight perturbation from an ideal single bond, they are significantly smaller than the other corresponding values reported for U=C bonds in chelated complexes which range from 0.22 to 0.52^58^. Therefore, the ellipticity data do not clearly corroborate with the outcome from the NBO and EDA-NOCV analyses. Perhaps, this might be due to the very polarized nature of the double dative bonds.

**Table 3:** QTAIM analysis of the complexes **2**, **4**, and **6**

Complex	BCP^a^	*ρ*(*r*_c_)^b^	∇^2^*ρ*(*r*_c_)^c^	*H*(*r*_c_)^d^	*ε*(*r*_c_)^e^
**2**	U-C1	0.079	0.112	−0.018	0.09
	U-N1	0.053	0.146	−0.005	
**4**	U-N2	0.050	0.139	−0.004	
	U-C1	0.075	0.107	−0.016	0.12
**6**	U-N	0.059	0.156	−0.006	
	U-C1	0.079	0.109	−0.018	0.11
	U-C2	0.015	0.046	0.008	

^a^The bond critical points

^b^The electron density (*ρ*(*r*_c_), au)

^c^Laplacian of electron density (∇^2^*ρ*(*r*_c_), au)

^d^Total electronic energy density (*H*(*r*_c_), au)

^e^Ellipticity of electron density (*ε*(*r*_c_))

## Discussion

We have prepared a set of complexes formed between CDP ligands and UCl_4_, which contain double dative bonds between carbon and uranium. Single-crystal X-ray diffraction analysis revealed a short uranium−carbon bond in these species. Theoretical calculations suggest that the nature of the bond between the carbon of CDP and uranium is a double dative bond. Both of the σ and π dative bonds in Cl_4_U⇇CDP are strongly polarized toward the carbon. Therefore, the concept of a double dative bond between carbon and *f*-block elements has been proposed. This finding shows that the carbones could serve as an effective ligand for the synthesis of *f*-block species with a double dative bond. These complexes reported herein further strengthen our understanding of the bonding between carbon and uranium, and thus the double dative bond might be a more general bonding motif in *f*-block chemistry. Our further studies will focus on the synthesis of other *f*-block species containing a double dative bond employ carbones as ligands, as well as investigations of their applications in small molecule activation.

## Methods

### General considerations

Experiments were performed under an N_2_ atmosphere using standard Schlenk-line and glove-box techniques. All solvents and reagents were dried and deoxygenated before use, using a solvent purification system. See the Supplementary Methods for detailed experimental procedures, crystallographic (Supplementary Tables 1-2), and computational analyses (Supplementary Dataset and Supplementary Tables 3-4).

### Preparation of **2**

NaHMDS (0.2 mL, 2 M in THF, 0.4 mmol) was added to an off-white suspension of ligand **1-(PF**_**6**_**)**_**2**_ (166 mg, 0.2 mmol) in THF. The resultant yellowish brown solution was stirred at RT for 2 h. Subsequently, a pre-cooled THF solution of UCl_4_ (76 mg, 0.2 mmol) was added. A yellowish green precipitate formed immediately upon addition of UCl_4_. The suspension was stirred for another 1 h and then filtered through Celite and washed with THF (5 mL × 3). The residue was then dissolved in dichloromethane and the resultant yellowish green solution was concentrated to ca. 5 mL and cooled at –35 °C overnight to afford yellow-green crystals. Once obtained as crystalline material, **2** is insoluble in aromatic and aliphatic solvents, and consequently a satisfactory ^13^C NMR spectrum was not available. However, in dichloromethane-d_2_, complex **2** decomposes slowly, allowing for ^1^H and ^31^P NMR spectroscopic measurement. Crystalline yield: 122 mg, 66%. ^1^H-NMR (CD_2_Cl_2_, 298 K, 400 MHz): δ 14.19 (s), 9.71 (s), 8.79 (s), −7.35 (s), and −41.54 (s) ppm. ^31^P{^1^H}-NMR (CD_2_Cl_2_, 298 K, 162.0 MHz): −215.87 (s) ppm. Anal. Calcd. for C_35_H_28_Cl_4_N_2_P_2_U: C 45.77; H 3.07; N 3.05. Found: C 45.91; H 3.03; N 3.01. FTIR ν/cm^−1^ (Nujol): 1573 (w), 1438 (s), 1216 (w), 1106 (s), 989 (m), 918 (w), 787 (w), 770 (w), 743 (s), 690 (s), 561 (w), 530 (m), 495 (m).

### Preparation of **4**

A white suspension of **3-(Br)**_**2**_ (141 mg, 0.2 mmol) in toluene (10 mL) was treated with 0.2 mL NaHMDS (2 M in THF, 0.4 mmol). The resultant yellow suspension was stirred at RT for 2 h and then filtered. The filtrate was concentrated to ca. 5 mL and a cold solution of UCl_4_ (76 mg, 0.2 mmol) in THF (5 mL) was added. The resultant mixture was stirred at RT for another 1 h before filtration, generating a yellowish green suspension. The residue was washed by toluene (5 mL × 3) and then dissolved in DCM. The green filtrate was concentrated and kept at −35 °C overnight to afford yellowish green micro crystalline product. The crystals were collected and washed with cold THF to afford complex **4** in 52% yield (95 mg). Once obtained as crystalline material from DCM, **4** is not soluble in aromatic and aliphatic solvents, and decomposes in polar solvents such as DCM. Alternatively, it was found that **4** could also crystalize from a mixture of deprotonated **3** and UCl_4_ in THF. Thus, the ^31^P NMR spectrum of **4** was attained by measuring the reaction mixture after 1 h in THF-d_8_. However, satisfactory ^1^H and ^13^C NMR spectra could not be obtained and a satisfactory electronic absorption spectrum is also not available for the same reason. ^31^P{^1^H}-NMR(THF-d_8_, 298 K, 162 MHz): δ –125.51 (d, *J* = 2 Hz), –155.13 ppm (d, *J* *=* 2.2 Hz). Anal. Calcd. for C_36_H_29_Cl_4_NP_2_U: C 47.13; H 3.19; N 1.53. Found: C 46.74; H 3.51; N 1.23. FTIR ν/cm^−1^ (Nujol): 1586 (w), 1439 (s), 1109 (m), 996 (w), 918 (s), 741 (s), 687 (m), 542 (w), 510 (w), 497 (w).

### Preparation of **6**

A white suspension of **5-(Br)**_**2**_ (140 mg, 0.2 mmol) in toluene (10 mL) was treated with 0.2 mL NaHMDS (2 M in THF, 0.4 mmol). The resultant yellow suspension was stirred at RT for 2 h before filtration. The filtrate was concentrated to ca. 5 mL and a cold solution of UCl_4_ (76 mg, 0.2 mmol) in THF (5 mL) was added. The resultant mixture was stirred at RT for another 1 h, generating white precipitates. The solvent was removed in vacuo and the yellowish green residue was washed with toluene (5 mL × 3) and then dissolved in DCM and filtered. The green filtrate was concentrated and kept at −35 °C overnight to afford yellowish green microcrystals. Those crystals were collected and washed with cold THF to afford complex **6** in 59% yield (109 mg). Once obtained as crystalline material from DCM, **6** is not soluble in common organic solvent, and decomposes in THF and DCM. Attempts to characterize **6** from reaction mixtures in THF-d_8_ failed to obtain reliable information. Therefore, no spectral is available for complex **6**. The reproducibility of the synthesis of complex **6** was assessed by solving and refining the data of single crystal collected from three independent syntheses. Those results were identical. Anal. Calcd. for C_37_H_30_Cl_4_P_2_U: C 48.49; H 3.30. Found: C 45.93; H 3.24. This complex consistently has low carbon content, possibly due to the high sensitivity. FTIR ν/cm^−1^ (Nujol): 1438 (s), 1109 (m), 996 (m), 918 (w), 797 (w), 736 (w), 686 (m), 559 (s), 542 (m), 499 (w).

## Electronic supplementary material

Supplementary Information

Supplementary Data

Description of Additional Supplementary Files

## Data Availability

The X-ray crystallographic coordinates for structures reported in this study have been deposited at the Cambridge Crystallographic Data Centre (CCDC), under deposition numbers CCDC-1850097 (**2**), 1850102 (**4**), and 1850103 (**6**). These data can be obtained free of charge from The Cambridge Crystallographic Data Centre via www.ccdc.cam.ac.uk/data_request/cif. The data that support the findings of this study are available from the corresponding author upon reasonable request.
