# Supplementary material for: Double dative bond between divalent carbon(0) and uranium
Source: Nat Commun. 2018 Nov 27;9:4997. doi: 10.1038/s41467-018-07377-6 (PMC6258733; doi:10.1038/s41467-018-07377-6)
Supplement: Supplementary file 1 — Supplementary Information [file 41467_2018_7377_MOESM1_ESM.pdf]

Double Dative Bond Between Divalent Carbon(0) and Uranium

Su et al.

Supplementary Figures

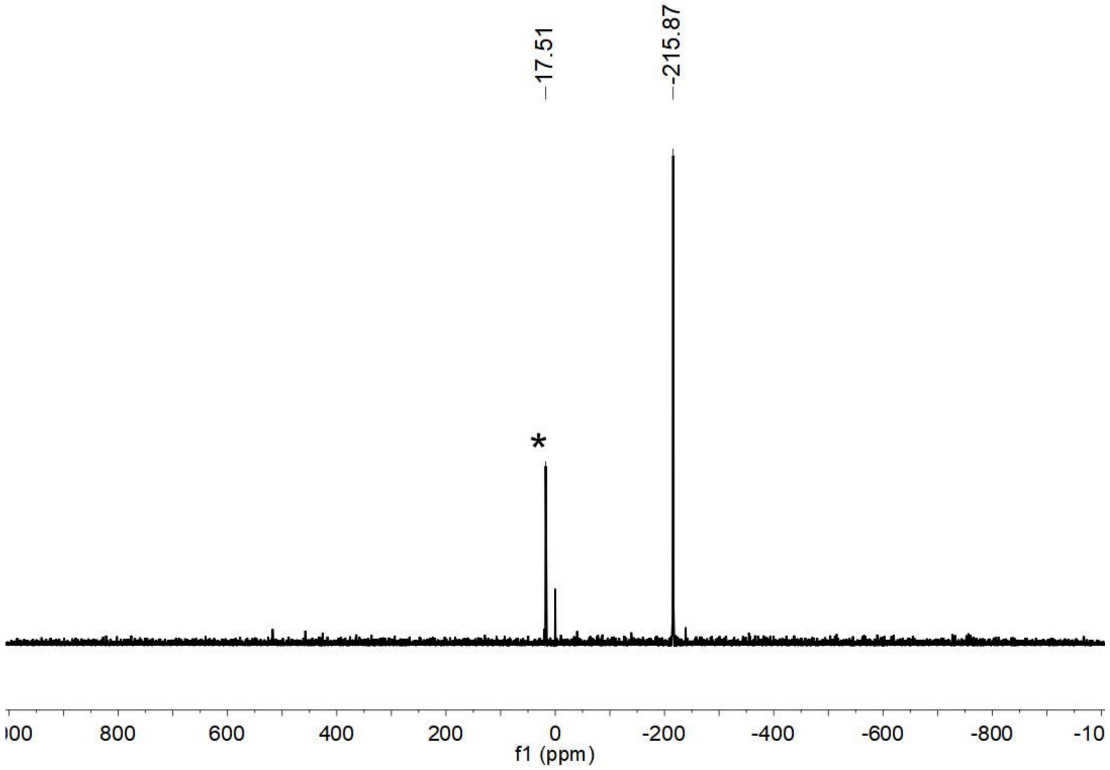

Supplementary Figure 1. $^{31}P\{^{1}H\}$-NMR spectrum of complex **2** in CD_2Cl_2. Asteriks are signals assigned to ligand precursor (**1**) due to decomposition of complex **2**.

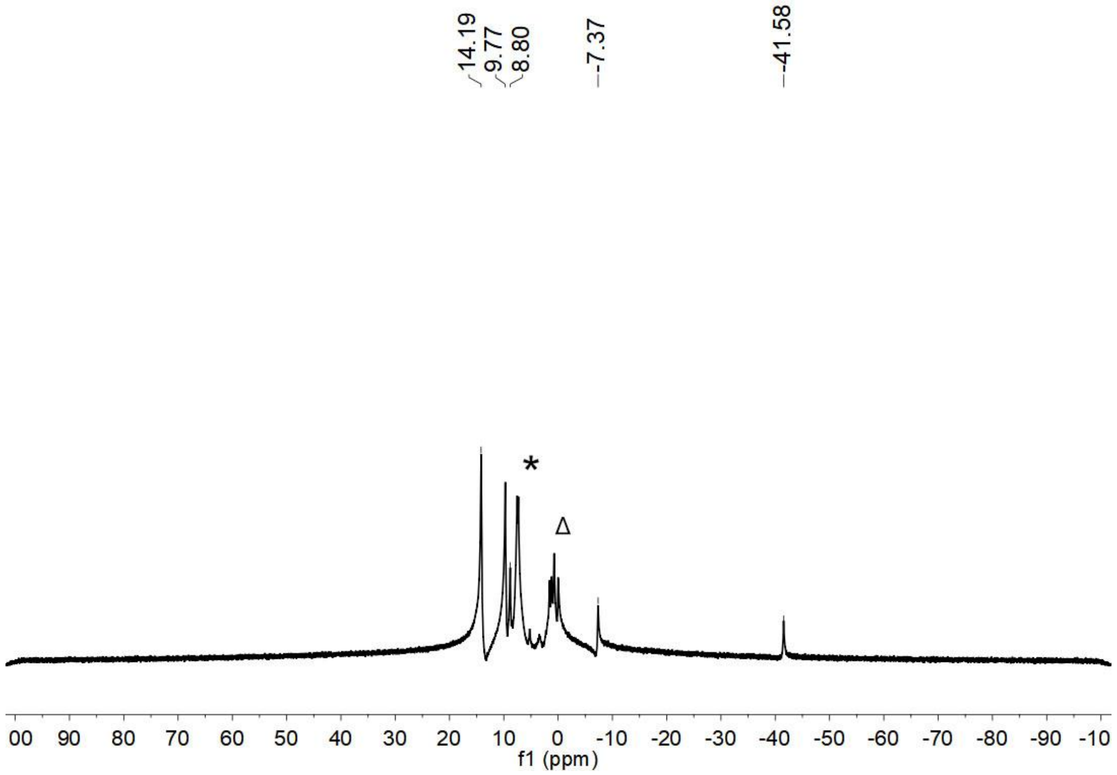

Supplementary Figure 2. ^{1}H-NMR spectrum of complex **2** in CD_2Cl_2. Asteriks are signals assigned to ligand precursor (**1**) due to decomposition of complex **2**. Triangle are singals of n-hexane.

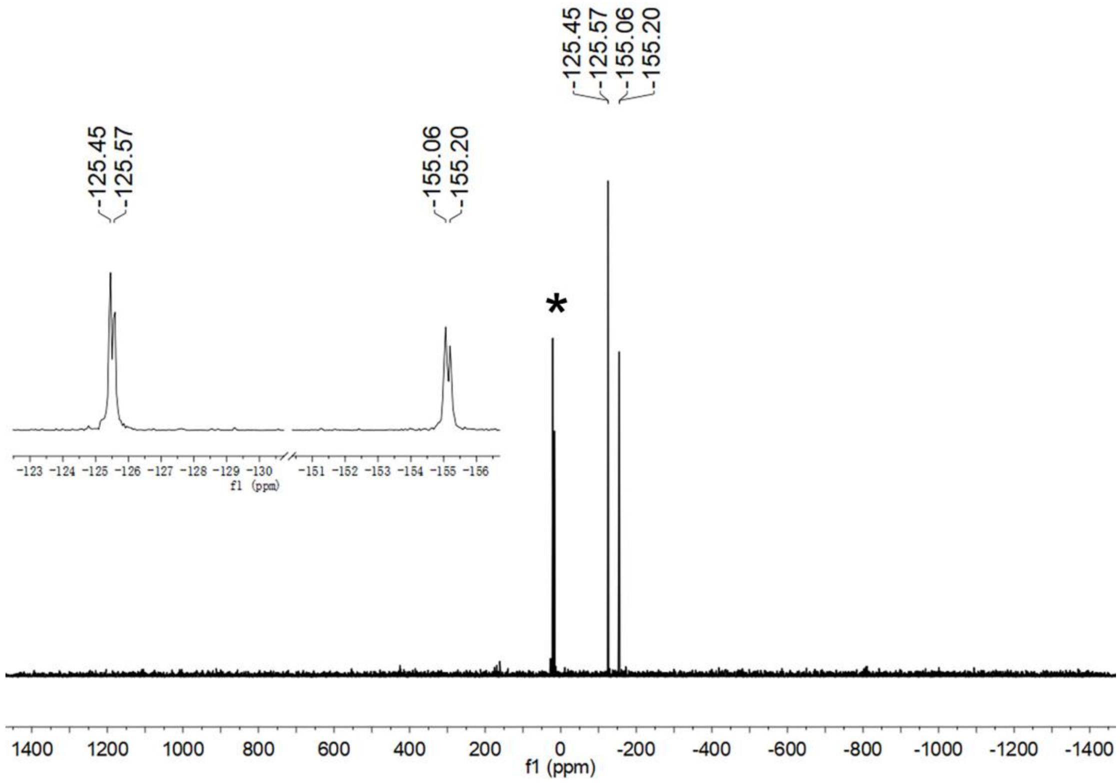

Supplementary Figure 3. $^{31}P\{^{1}H\}$-NMR spectrum of complex **4** in THF-d_8. Asteriks are signals assigned to ligand precursor (**3**) due to decomposition of complex **4**.

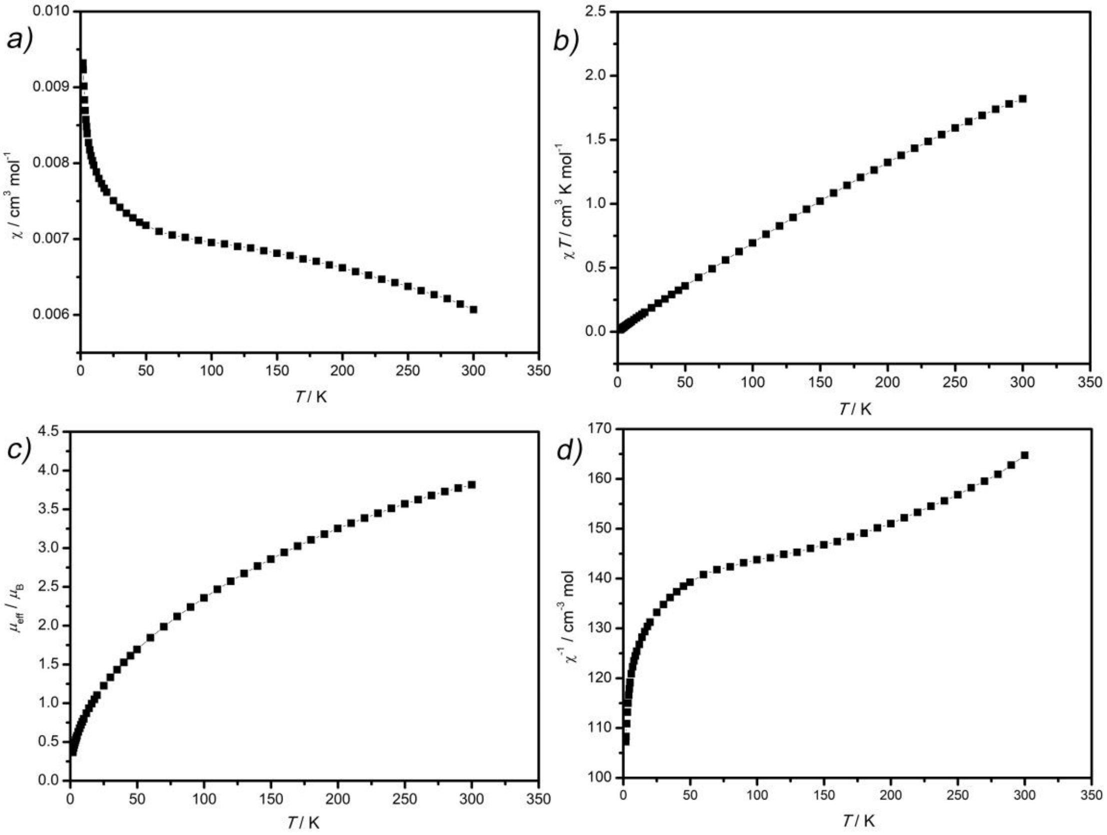

Supplementary Figure 4. Variable-Temperature SQUID Magnetisation for **2**, presented as: (a) χ vs T; (b) χT vs T; (c) μ_{eff} vs T; (d) χ^{-1} vs T.

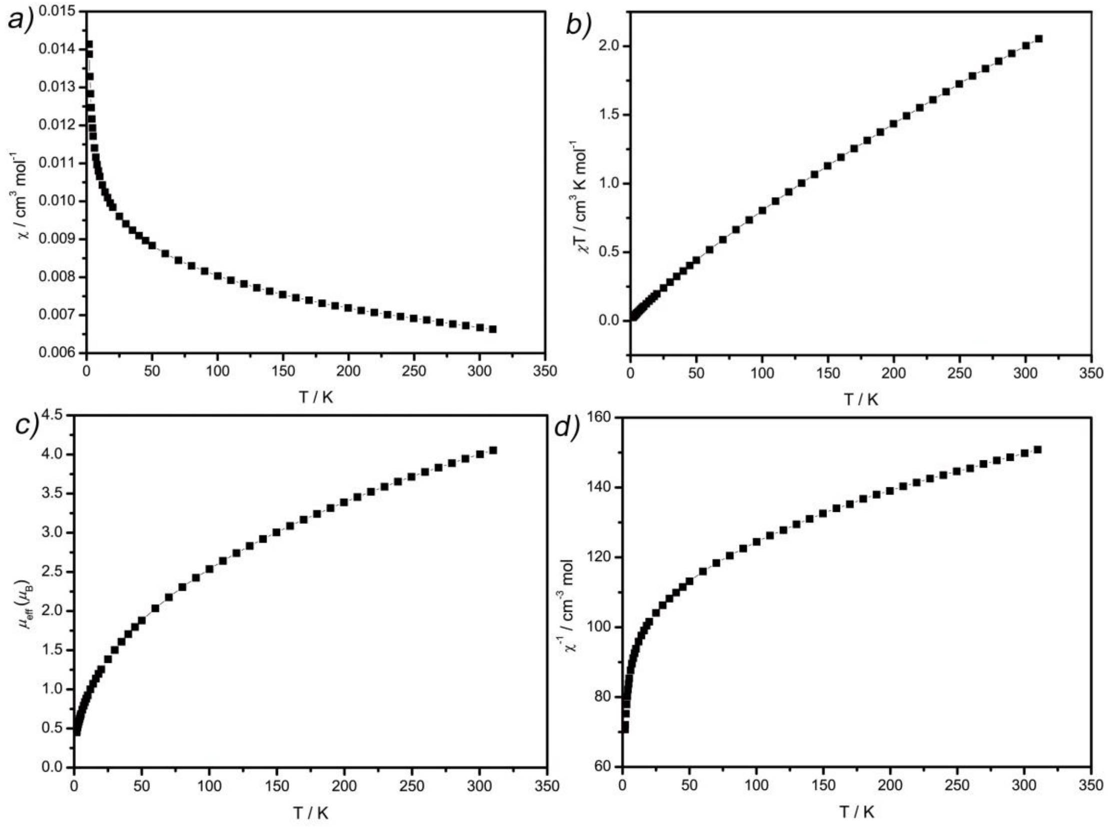

Supplementary Figure 5. Variable-Temperature SQUID Magnetisation for **4**, presented as: (a) χ vs T; (b) χT vs T; (c) μ_{eff} vs T; (d) χ^{-1} vs T.

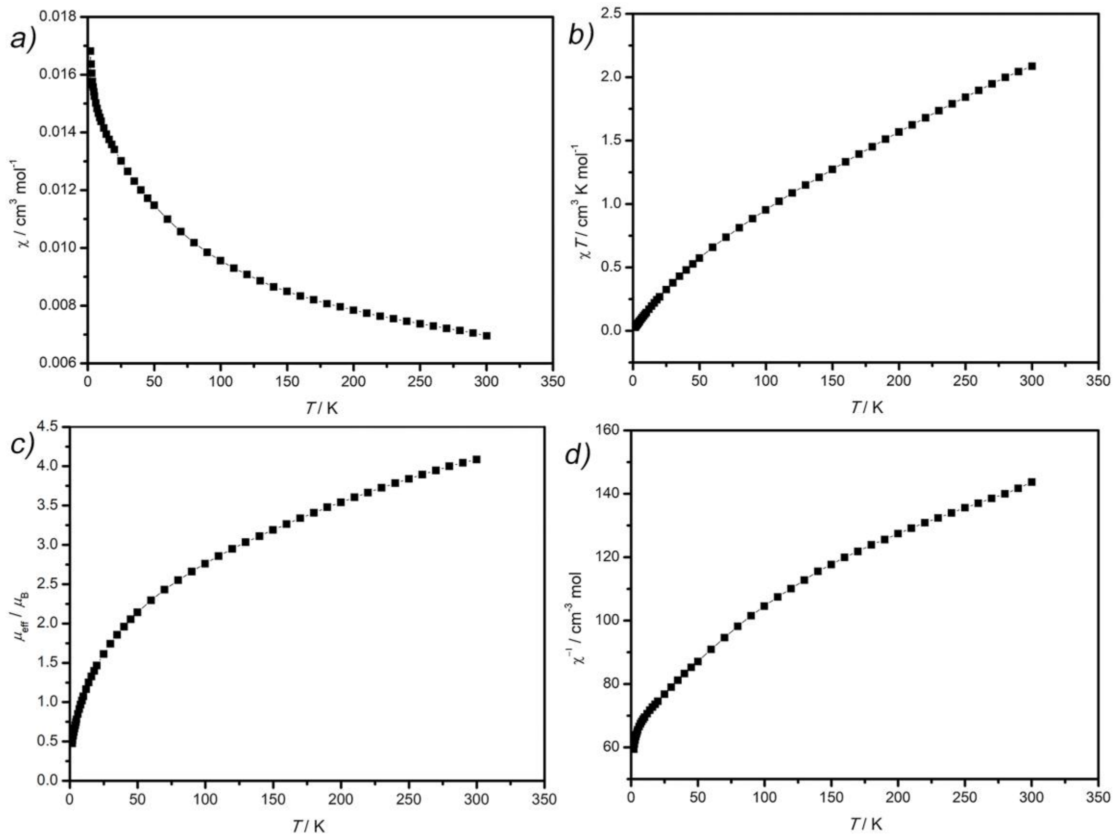

Supplementary Figure 6. Variable-Temperature SQUID Magnetisation for **6**, presented as: (a) χ vs T; (b) χT vs T; (c) μ_{eff} vs T; (d) χ^{-1} vs T.

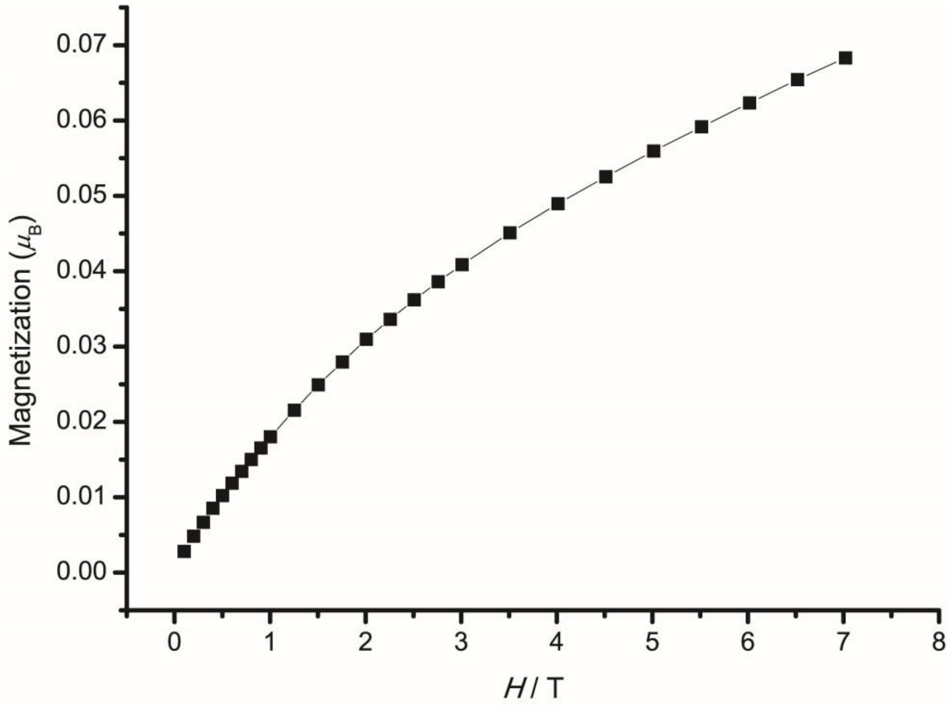

Supplementary Figure 7. Field-dependent SQUID magnetization for complex **2** at 1.8 K.

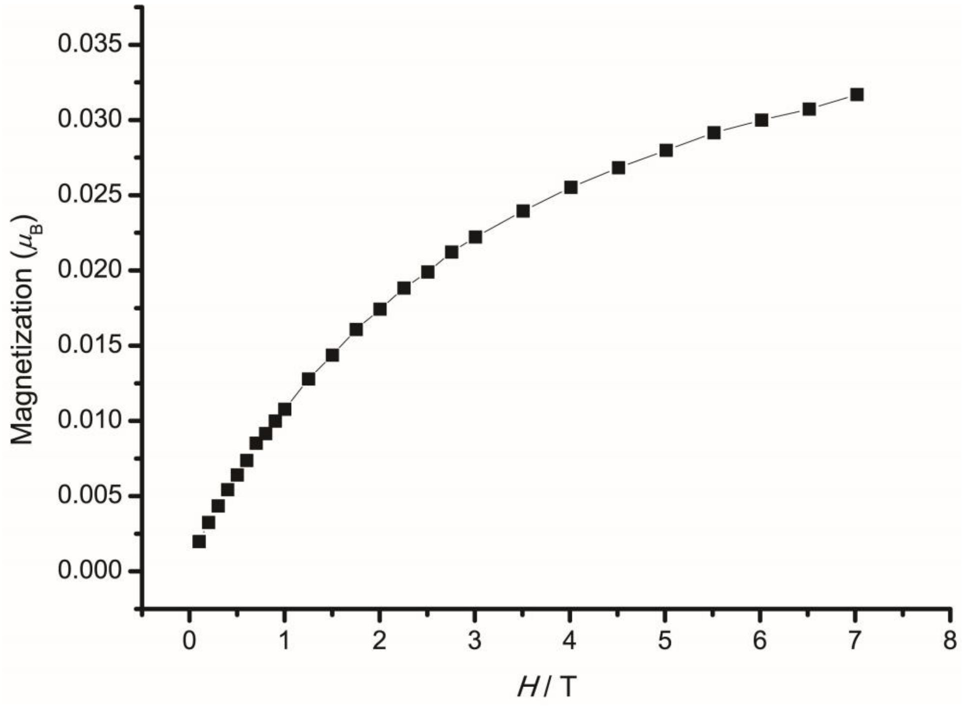

Supplementary Figure 8. Field-dependent SQUID magnetization for complex **4** at 1.8 K.

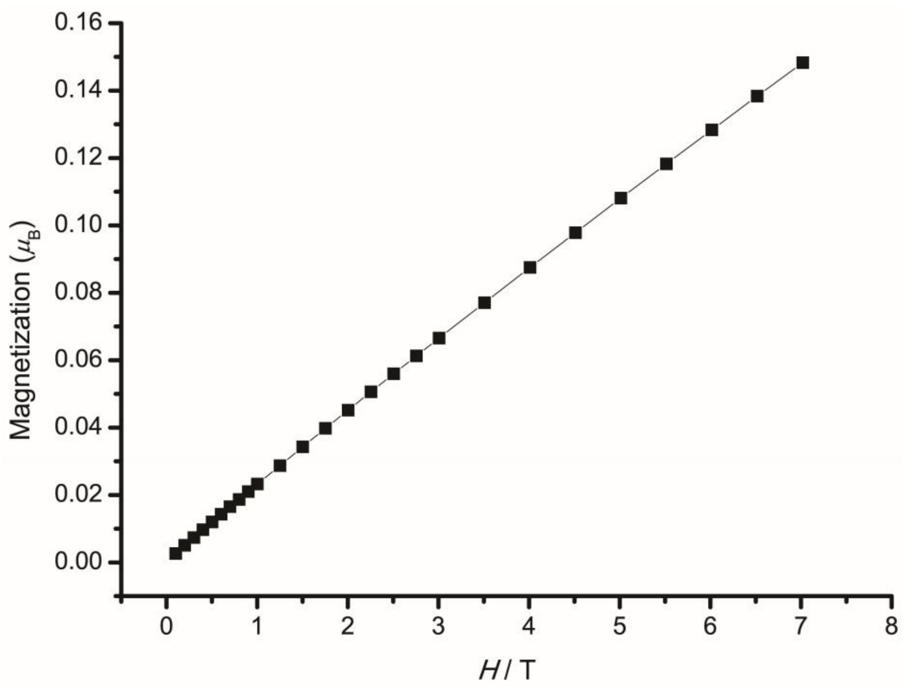

Supplementary Figure 9. Field-dependent SQUID magnetization for complex **6** at 1.8 K.

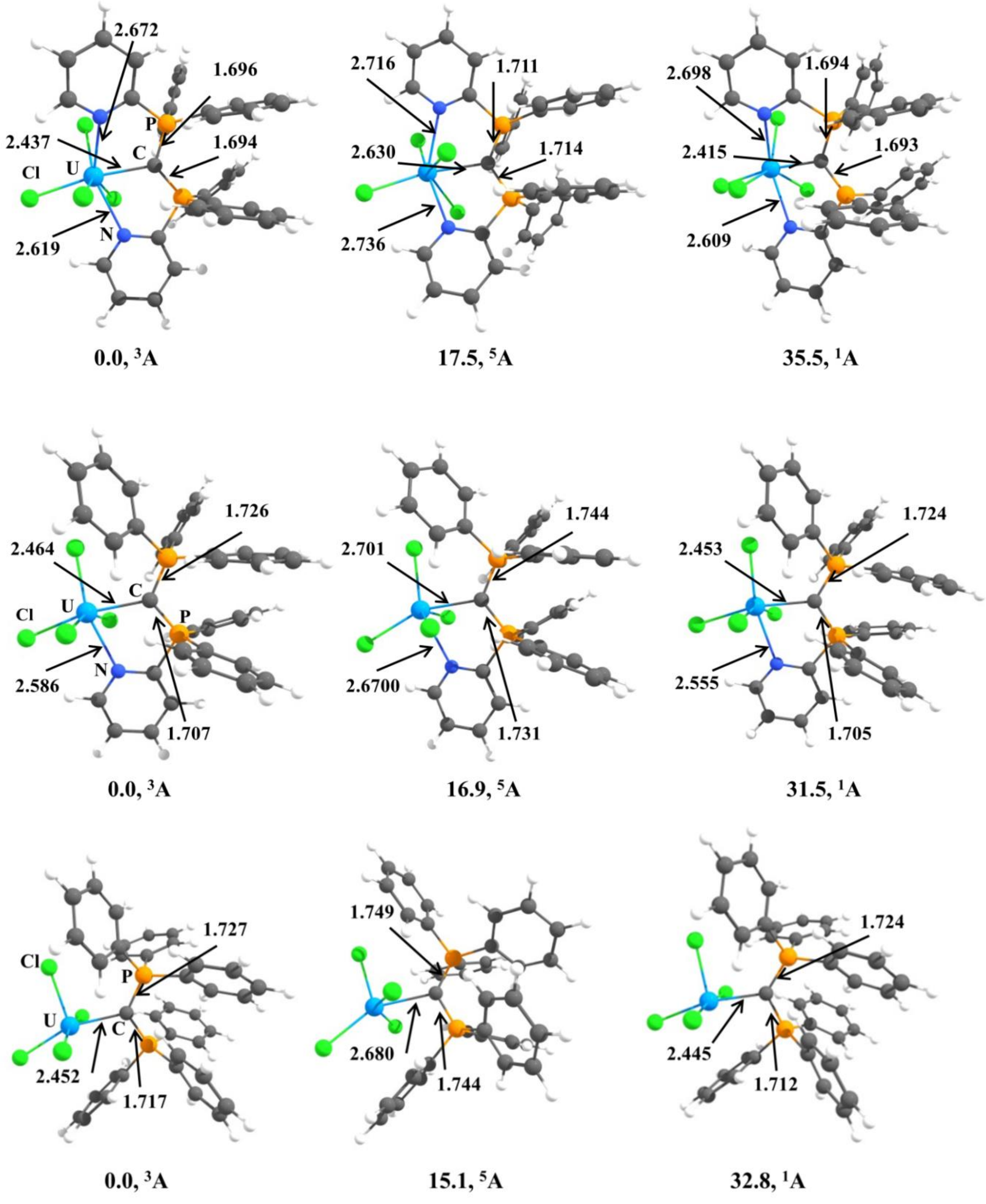

Supplementary Figure 10. The optimized structure of **2**, **4** and **6** complexes with different spin states at the BP86-D3(BJ)/def2-SVP/Stuttgart RLC ECP level (Stuttgart RLC ECP for U and def2-SVP for other elements). Relative energies are in kcal mol^{-1}. Distances are in Å.

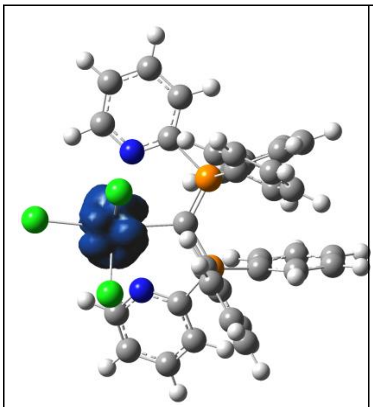<br>**2**<br>**(2.04)**	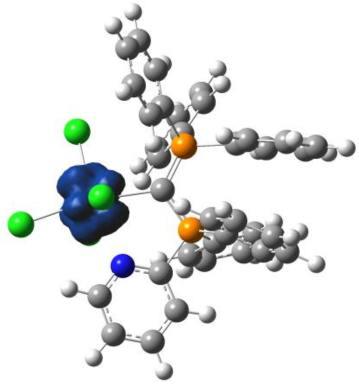<br>**4**<br>**(2.07)**	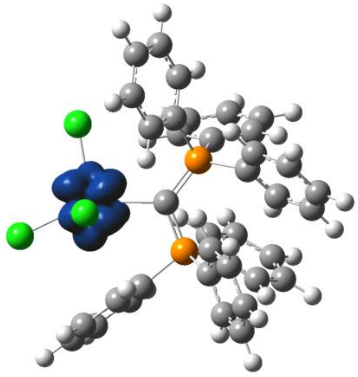<br>**6**<br>**(2.14)**

Supplementary Figure 11. The plot of the total spin density in triplet **2**, **4**, and **6** complexes. The NBO spin density on U is given in parentheses. The isovalue is 0.005.

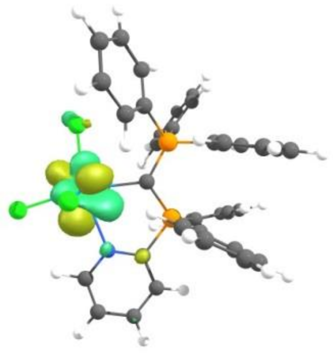<br>**SOMO**<br>**ε_{α} = -3.54**	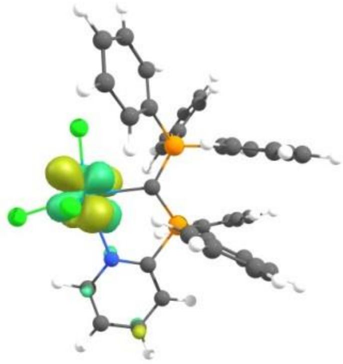<br>**SOMO-1**<br>**ε_{α} = -3.62**	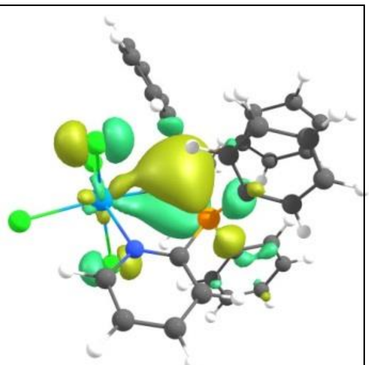<br>**HOMO**<br>**$\varepsilon_{\alpha}/\varepsilon_{\beta}$ = -5.30/-5.30**
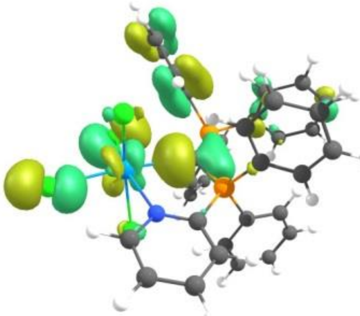<br>**HOMO-1**<br>**$\varepsilon_{\alpha}/\varepsilon_{\beta}$ = -6.09/-6.02**	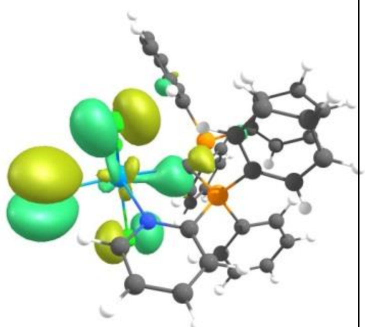<br>**HOMO-2**<br>**$\varepsilon_{\alpha}/\varepsilon_{\beta}$ = -6.16/-6.15**	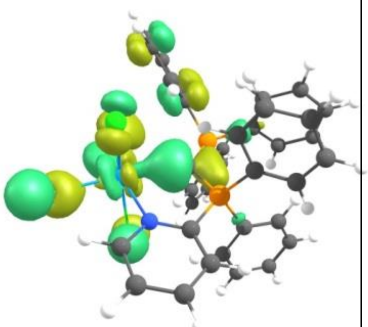<br>**HOMO-3**<br>**$\varepsilon_{\alpha}/\varepsilon_{\beta}$ = -6.24/-6.20**

Supplementary Figure 12. Some selective molecular orbitals of triplet **4** complex depicting the odd electrons and U-C interaction. The MO energy eigenvalues are given in eV. The isosurface value is 0.03 e Å^{-3}.

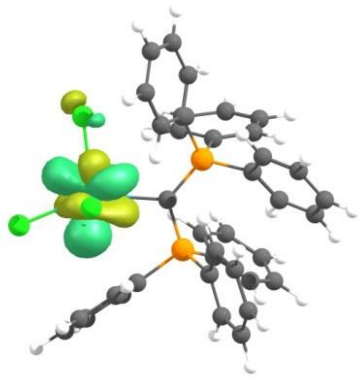<br>**SOMO**<br>ε_{α} **= -3.59**	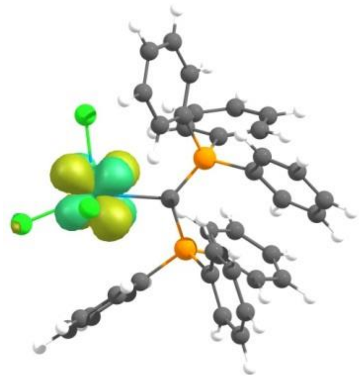<br>**SOMO-1**<br>ε_{α} **= -3.76**	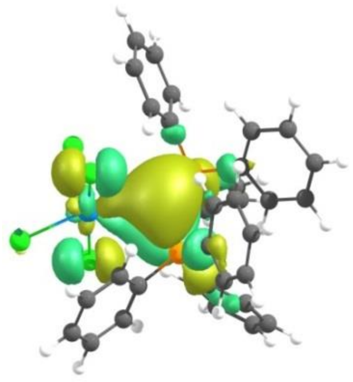<br>**HOMO**<br>$\varepsilon_{\alpha}/\varepsilon_{\beta}$ **= -5.35/-5.36**
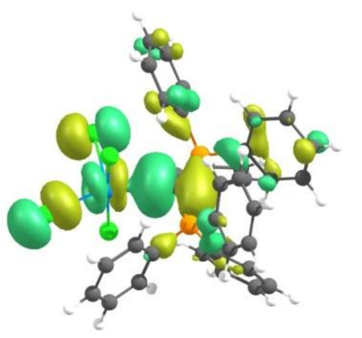<br>**HOMO-1**<br>$\varepsilon_{\alpha}/\varepsilon_{\beta}$ **= -6.09/-6.01**	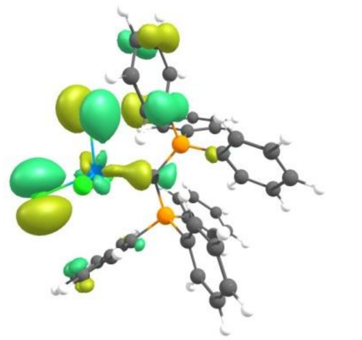<br>**HOMO-4**<br>$\varepsilon_{\alpha}/\varepsilon_{\beta}$ **= -6.40/-6.38**	

Supplementary Figure 13. Some selective molecular orbitals of triplet **6** complex depicting the odd electrons and U-C interaction. The MO energy eigenvalues are given in eV. The isosurface value is 0.03 e Å^{-3}.

	NBO	Contribution from atoms to the orbitals	Atomic orbitals
**2**	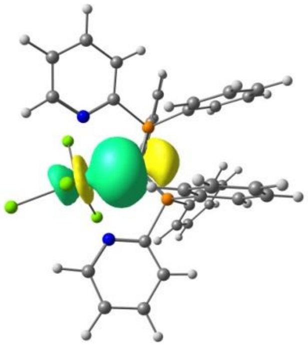<br>U-C σ-bond ON = 1.82 e^-	U(17.0%)-C(83.0%)	U: s(8.8%) p(0.5%) d(39.0%) f(51.7%)<br><br>C: s(32.9%) p(67.0%)
	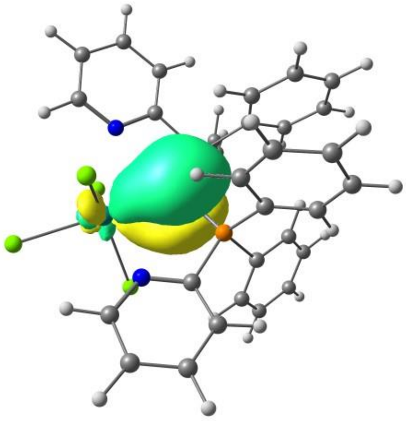<br>U-C π-bond ON = 1.64 e^-	U(9.3%)-C(90.7%)	U: s(0.1%) p(0.1%) d(45.5%) f(54.3%)<br><br>C: s(0.3%) p(99.5%)
**4**	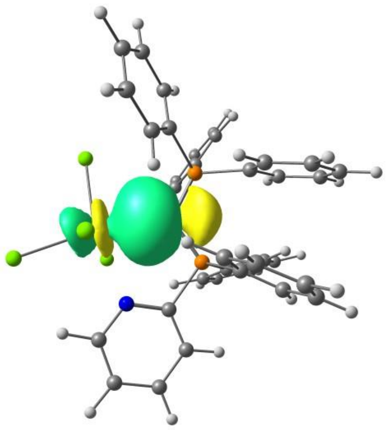<br>U-C σ-bond ON = 1.84 e^-	U(16.6%)-C(83.4%)	U: s(11.0%) p(0.4%) d(39.2%) f(49.4%)<br><br>C: s(33.9%) p(65.9%)

	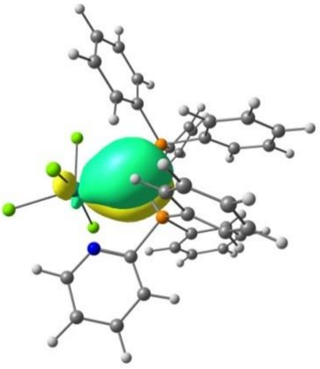 U-C π-bond ON = 1.65 e^-	U(9.2%)-C(90.8%)	U: s(0.0%) p(0.1%) d(46.8%) f(53.1%)<br><br>C: s(0.3%) p(99.5%)
**6**	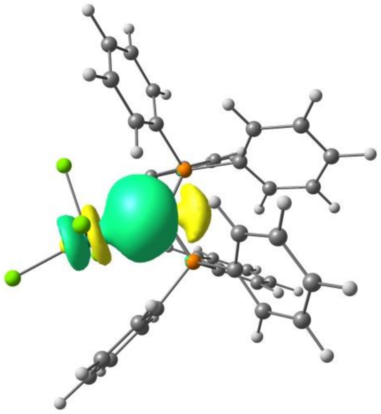 U-C σ-bond ON = 1.84 e^-	U(16.3%)-C(83.7%)	U: s(10.7%) p(0.4%) d(42.3%) f(46.7%)<br><br>C: s(33.3%) p(66.6%)
	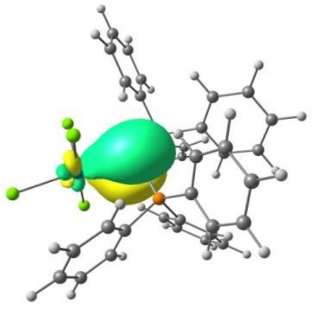 U-C π-bond ON = 1.66 e^-	U(10.9%)-C(89.1%)	U: s(0.1%) p(0.0%) d(45.2%) f(54.7%)<br><br>C: s(0.1%) p(99.7%)

Supplementary Figure 14. The results of NBO analysis.

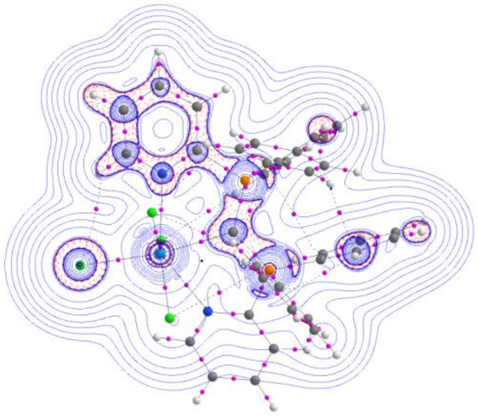

2 (at C1-U-N2 plane)

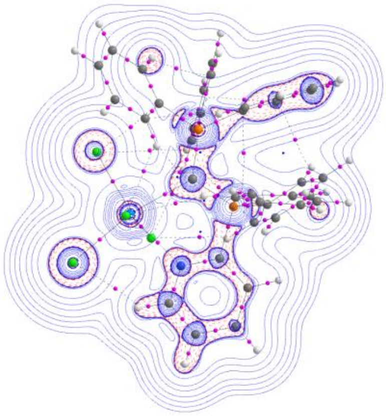

4 (at C1-U-N plane)

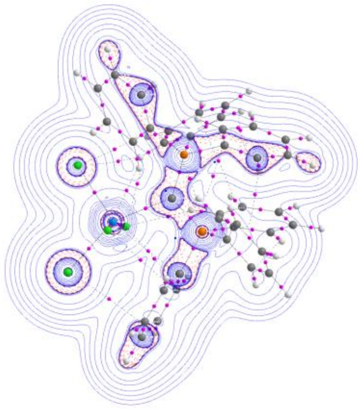

6 (at C1-U-C2 plane)

Supplementary Figure 15. Plot of Laplacian $\nabla^2\rho(r)$ of electron density and bond paths at the BP86-D3(BJ)/def2-TZVPP/SARC-ZORA//BP86-D3(BJ)/def2-TZVPP/ Stuttgart RSC ECP level. The purple balls are bond critical points (BCPs). Ring critical points (RCPs) are not shown for the sake of clarity. In all cases, there is a large area of charge concentration at carbon pointing to the uranium center along the U-C1 bond path. This can be interpreted as a donation of the two lone-pair electrons on the carbon atoms towards uranium. Similarly, an area in which charge is accumulated at nitrogen is also pointed toward the uranium along U-N bond path in **2** and **4**. A bond path and critical point are also observed in complex **6** between U and C2 of the –Ph ring.

Supplementary Methods

1. Experimental procedures: Synthesis of uranium complexes was carried out in glove-box (SG1800/750TS-F, VIGOR) under an N_2 atmosphere with oxygen and water contents less than 1 ppm. The solvents were obtained by passing through a Solv Purer G5 (MIKROUNA) solvent purification system and further dried over 4 Å molecular sieves. Deuterated solvents were dried over Na/K (THF-d_8 and bezene-d_6) or distilled from CaH_2 (DCM-d_2) and stored under an N_2 atmosphere prior to use. Other reagents were used as received without further purification. The nuclear magnetic resonance spectroscopy was recorded on a Bruker AVIII-400 (^{1}H 400.1 MHz; ^{13}C 100.6 MHz; ^{31}P 162.0 MHz) spectrometer at room temperature. ^{1}H and ^{13}C NMR chemical shifts are referred to tetramethylsilane, and ^{31}P NMR chemical shifts are relative to 85% H_3PO_4. The absolute values of the coupling constants are given in Hertz (Hz). Multiplicities are abbreviated as singlet (s), doublet (d), triplet (t), multiplet (m), and broad (br). Elemental analyses (C, H, N) were performed on a Vario EL III elemental analyser at Shanghai Institute of Organic Chemistry, the Chinese Academy of Sciences. FT-IR was performed with a Bio-Rad FTS-185 spectrometer. Magnetic properties were measured on MPMS SQUID VSM (Quantum Design). Ligands **3** and **5** and UCl_4 were prepared according to published methods[1-3].

Synthesis of ligand 1-(PF_6)$_2$.

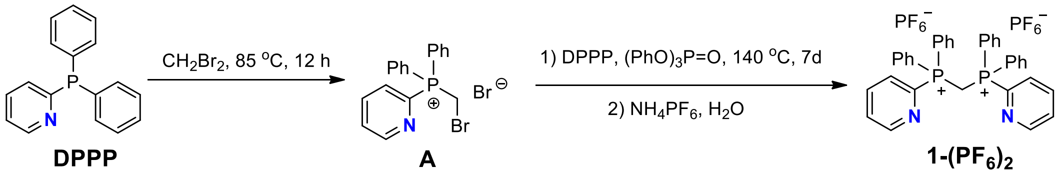

Compound A: A solution of diphenyl-(2-pyridinyl)phosphine (**DPPP**) (5.20 g, 20.0

mmol) in dibromomethane (200 mmol) was heated at 85 ^{o}C overnight, generating a yellowish green suspension. The suspension was filtered, the residue was washed with toluene (30 ml × 3) and redissolved in MeOH and recrycstallized from EtOAc. The white precipitate was dried *in vacuo* to afford the desired product. Yield: 5.98 g (69 %). ^{1}H-NMR (DMSO-d_6, 298 K, 400 MHz): *δ* 9.03 (d, *J* = 4.1 Hz, 1H), 8.18–8.29 (m, 2H), 7.66–8.11 (m, 11H), 5,67 (d, *J* = 6.9 Hz, 2H) ppm. ^{13}C-NMR (DMSO-d_6, 298 K, 100 MHz): δ 152.7 (d, *J* = 19.8 Hz), 143.6 (d, *J* = 119.9 Hz), 138.9 (d, *J* = 10.2 Hz), 136.0 (d, *J* = 2.9 Hz), 134.8 (d, *J* = 10.0 Hz), 132.2 (d, *J* = 24.2 Hz), 130.7 (d, *J* = 12.8 Hz), 129.3 (d, *J* = 3.4 Hz), 116.8 (d, *J* = 88.1 Hz), 16.0 (d, *J* = 53.8 Hz) ppm. ^{31}P{^{1}H}-NMR (DMSO-d_6, 298 K, 162 MHz): δ 18.70 (s) ppm.

Ligand 1-(PF_6)$_2$: A mixture of compound **A** (1.75 g, 4.00 mmol), diphenyl-(2-pyridinyl)phosphine (2.10 g, 8.00 mmol) and triphenyl phosphonate (5.00 g) in a 100 mL Schlenk flask was evacuated for 5 min, and then heat at 140 ^{o}C for 7 days. Addition of toluene (50 mL) into the resulting dark wax gave a grey suspension which was stirred for 1 h. It was then filtered and the residue was redissolved in H_2O to give a brown solution. Addition of NH_4PF_6 into this solution resulted in formation of an off-white precipitates. The precipitates was washed with cold MeOH and filtered, then the grey solid was dried under vacuum to afford ligand **1-(PF_6)$_2$**. Yield: 300 mg (10 %). ^{1}H-NMR (DMSO-d_6, 298 K, 400 MHz): *δ* 8.62 (d, *J* = 4.4 Hz, 2H), 8.03–7.85 (m, 4H), 7.72–7.37 (m, 24H). ^{13}C-NMR (DMSO-d_6, 298 K, 100 MHz): δ 152.10 (s), 151.0 (t, *J* = 10 Hz), 150.8 (s), 137.8 (t, *J* = 4 Hz), 133.5 (t, *J* = 6 Hz), 133.3 (s), 129.5 (t, *J* = 6 Hz), 129.3 (s), 126.9 (s), 125.9 (s), –5.19 (t, J = 120.9 Hz).

$^{31}P\{^1H\}$-NMR (DMSO-d_6, 298 K, 162 MHz): δ 17.25 (s, *P*Ph_2Py), –144.20 ppm (septet, *P*F_6).

2. X-ray crystallographic analysis

Crystals suitable for X-ray diffraction were grown from a solution of CDP-UCl_4 complexes in DCM at -35 ^oC. The intensity data were collected with a Bruker APEX-II CCD area detector using graphite-monochromated Mo Kα radiation (λ = 0.71073 Å). Multiscan or empirical absorption corrections (SADABS) were applied. The structures were solved by Patterson methods, expanded by difference Fourier syntheses, and refined by full-matrix least squares on F^2 using the Bruker SHELXTL-2014 program package [4]. All non-hydrogen atoms were refined anisotropically. Hydrogen atoms were introduced at their geometric positions and refined as riding atoms. The X-ray crystal structures have been deposited in the Cambridge Crystallographic Data Centre (CCDC). The data can be obtained free of charge from the CCDC (www.ccdc.cam.ac.uk/data_request/cif). Details of the data collection and refinement for complexes **2**, **4**, and **6** are given in Table S1 and Table S2.

3. Theoretical calculation

Computational details: Density functional theory (DFT) calculations were performed to elucidate the bonding situation in **2**, **4**, and **6**. The geometries of these species in their singlet, triplet and quintet spin states were firstly optimized at BP86-D3(BJ)[5-8]/def2-SVP[9]/Stuttgart RLC ECP level, where Stuttgart RLC ECP basis set (includes ECP for 78 core electrons, large core) is used for U and def2-SVP for

other elements. The most stable triplet complexes of **2**, **4**, and **6** were further reoptimized by using the larger basis set def2-TZVPP[10,11] and Stuttgart RSC ECP[12] (includes ECP for 60 core electrons), which is denoted as BP86-D3(BJ)/def2-TZVPP/Stuttgart RSC ECP level. The nature of the stationary points and the thermodynamic corrections were also obtained at this level of theory. The partial charges were computed via natural bond orbital (NBO) analysis by using NBO 6.0 program[13]. All these calculations were carried out by using Gaussian 09 software package[14].

The analysis of the electron density with the QTAIM method[15] was performed at the BP86-D3(BJ)/def2-TZVPP/SARC-ZORA//BP86-D3(BJ)/def2-TZVPP/Stuttgart RSC ECP level with the AIMAll program package[16], where all electron SARC-ZORA basis set is used for U instead of ECP. The nature of the bonding was investigated by means of an energy decomposition analysis (EDA)[17] together with the natural orbitals for chemical valence (NOCV)[18,19] method by using the ADF 2017.01program package[20,21].

The EDA-NOCV[22,23] calculations were carried out at the BP86-D3(BJ)/TZ2P[24]//BP86-D3(BJ)/def2-TZVPP/Stuttgart RSC ECP level where the spin-orbit relativistic effects were considered for U using the zeroth-order regular approximation (ZORA). The bonding analysis focuses on the interaction energy (ΔE_{int}) between two fragments, which can be decomposed into four main components (Supplementary Equation 1).

$$\Delta E_{\mathrm{int}} = \Delta E_{\mathrm{elstat}} + \Delta E_{\mathrm{Pauli}} + \Delta E_{\mathrm{disp}} + \Delta E_{\mathrm{orb}} \quad (1).$$

The term $\Delta E_{\mathrm{elstat}}$ corresponds to the quasiclassical electrostatic interaction between the unperturbed charge distributions of the prepared fragments and is mostly attractive. The Pauli repulsion $\Delta E_{\mathrm{Pauli}}$ is the energy change associated with the transformation from the superposition of the unperturbed electron densities of the isolated fragments to the wavefunction, which properly obeys the Pauli principle through explicit antisymmetrization and renormalization of the production wavefunction. The term ΔE_{disp} represents the dispersion interaction between the fragments. The term ΔE_{orb} originates from the mixing of orbitals, charge transfer and polarization between the isolated fragments, which can be further decomposed into contributions from each irreducible representation of the point group of the interacting system (Supplementary Equation 2).

$$\Delta E_{orb} = \sum_{r} \Delta E_{r} \qquad (2)$$

The EDA-NOCV method combines the energy decomposition analysis (EDA) together with the natural orbitals for chemical valence (NOCV), which makes it possible to partition the total orbital interactions into pairwise contributions of the orbital interactions. The charge deformation $\Delta\rho_k(r)$, which comes from the mixing of the orbital pairs $\psi_{\mathrm{k}}(\mathrm{r})$ and $\psi_{\mathrm{-k}}(\mathrm{r})$ of the interacting fragments in the compound gives the amount and the shape of the charge flow due to the orbital interactions (Supplementary Equation 3). The associated energy term ΔE_{orb} gives the strength of the orbital interactions (Supplementary Equation 4). Therefore, an EDA-NOCV analysis provides both qualitative ($\Delta\rho_{orb}$) and quantitative (ΔE_{orb}) information of the strength of individual pairs of orbital interactions. The novel EDA-NOCV method is

in our view the most advanced partitioning method that provides deep insight into the nature of chemical bonding. Further details on the EDA method and its application to the analysis of the chemical bond can be found in the literature[25,26].

$$\Delta\rho_{orb}(r) = \sum_k \Delta\rho_k(r) = \sum_{k=1}^{N/2} \nu_k [-\psi_{-k}^2(r) + \psi_k^2(r)] \qquad (3)$$

$$\Delta E_{orb} = \sum_k \Delta E_k^{orb} = \sum_{k=1}^{N/2} \nu_k [-F_{-k,-k}^{TS} + F_{k,k}^{TS}] \qquad (4)$$

Supplementary Tables

Supplementary Table 1. Crystal data and structure refinements for **2** and **4**.

Complex	**2**·2CH_2Cl_2	**4**
Formula	$C_{37}H_{32}Cl_8N_2P_2U$	$C_{36}H_{29}Cl_4NP_2U$
*M*r [g/mol]	1088.21	917.37
Temp. [K]	153(2)	153(2)
Wavelength [Å]	0.71073	
Crystal system	triclinic	
Space group	P-1	
a [Å]	9.9480(6)	10.3828(7)
b [Å]	10.5619(7)	12.5869(9)
c [Å]	19.6614(12)	16.1499(12)
α [°]	98.501(2)	68.099(2)
β [°]	91.551(2)	79.671(2)
γ [°]	105.356(2)	84.990(2)
Volume [$Å^3$]	1965.4(2)	1926.0(2)
Z	2	
$D_{calcd.}$ [g/cm^3]	1.839	1.582
μ(MoKα) (mm^{-1})	4.786	4.598
F(000)	1052.0	884.0
Θ_{max} (°)	25	

Data/parameters	6898/451	6729/397
Final R indices (I> 2σ(I))	R1 = 0.0424<br>wR2 = 0.0917	R1 = 0.0378<br>wR2 = 0.0849
R indices (all data)	R1 = 0.0490<br>wR2 = 0.0943	R1 = 0.0495<br>wR2 = 0.0879
GOF	1.122	1.066
Largest diff. peak /hole (e ·Å^3)	1.51/-1.13	1.14/-1.03
CCDC	1850097	1850102

Supplementary Table 2. Crystal data and structure refinements for **6** from three independent syntheses.

Complex	**6** ·1.5CH_2Cl_2		
Formula	$C_{38.5}H_{33}Cl_7P_2U$		
*M*r [g/mol]	1043.77		
Temp. [K]	153(2)	153(2)	153(2)
Wavelength [Å]	0.71073		
Crystal system	triclinic		
Space group	P-1		
a [Å]	11.5198(6)	11.5016(6)	11.5252(7)
b [Å]	11.7902(6)	11.7868(7)	11.7979(7)
c [Å]	17.2840(10)	17.2716(9)	17.2853(10)
α [°]	76.952(2)	76.943(2)	76.936(2)
β [°]	75.215(2)	75.1820(10)	75.198(2)
γ [°]	61.3690(10)	61.3060(10)	61.331(2)
Volume [$Å^3$]	1977.39(19)	1970.88(19)	1978.9(2)
Z	2		
$D_{calcd.}$ [g/cm^3]	1.753	1.759	1.752
μ(MoKα) (mm^{-1})	4.598	4.701	4.682
F(000)	1010.0		
Θ_{max} (°)	25		
Data/parameters	6922/451	6907/451	6888/451

Final R indices (I> 2σ(I))	R1 = 0.0204 wR2 = 0.0498	R1 = 0.0250 wR2 = 0.0608	R1 = 0.0747 wR2 = 0.2250
R indices (all data)	R1 = 0.0224, wR2 = 0.0505	R1 = 0.0291 wR2 = 0.0620	R1 = 0.0769 wR2 = 0.2289
GOF	1.033	1.090	1.057
Largest diff. peak /hole (e ·Å^{3})	0.48/-0.67	0.60/-0.61	7.40/-7.66
CCDC	1850103	1856520	1856521

Supplementary Table 3. The U=C stretching frequencies of the studied complexes at the BP86-D3(BJ)/def2-TZVPP/Stuttgart RSC ECP level.

**Complex**	**ν(U=C), cm^{-1}**
**2**	674
**4**	673
**6**	671

Supplementary Table 4. The partial natural charges on some selective atomic centers and fragments (*q* in au), Wiberg bond order (WBO) at the BP86-D3(BJ)/def2-TZVPP/Stuttgart RSC ECP level and Nalewajski-Mrozek bond order (NMBO) computed from two-electron valence indices based on partitioning of $Tr(\Delta P)^2$ (4-index set) at the BP86-D3(BJ)/TZ2P-ZORA level.

Complex	*q*						NMBO		WBO	
	U	C1	P1	P2	UCl_4	CDP	U-C1	U-N	U-C1	U-N
**2**	0.77	-1.39	1.60	1.62	-0.64	+0.64	1.30	0.57, 0.59	0.66	0.32, 0.34
**4**	0.87	-1.41	1.60	1.62	-0.52	+0.52	1.34	0.67	0.66	0.37
**6**	0.87	-1.43	1.62	1.62	-0.50	+0.50	1.41	-	0.69	-

Supplementary References

1. Alcarazo, M. Radkowski, K. Mehler, G. Goddard, R. Furstner, A. Chiral heterobimetallic complexes of carbodiphosphoranes and phosphinidene-carbene adducts. *Chem. Commun.* **49**, 3140-3142 (2013).

2. Kiplinger, J. L. Morris, D. E. Scott, B. L. Burns, C. J. Convenient Synthesis, Structure, and Reactivity of $(C_5Me_5)U(CH_2C_6H_5)_3$: A Simple Strategy for the Preparation of Monopentamethylcyclopentadienyl Uranium(IV) Complexes. *Organometallics*, **21**, 5978-5982 (2002).

3. Zybill, C. Mueller, G. Mononuclear complexes of copper(I) and silver(I) featuring the metals exclusively bound to carbon. Synthesis and structure of (.eta.5-pentamethylcyclopentadienyl)[(triphenylphosphonio)(triphenylphosphoranylidene)methyl]copper(I). *Organometallics*, **6**, 2489-2494 (1987).

4. Sheldrick, G. M. *SHELXT* – Integrated space-group and crystal-structure determination. *Acta Cryst.* C**71**, 3–8 (2015).

5. Becke, A. D. Density-functional exchange-energy approximation with correct asymptotic behavior. *Phys. Rev. A*, **38**, 3098-3100 (1988).

6. Perdew, J. P. Density-functional approximation for the correlation energy of the inhomogeneous electron gas. *Phys. Rev. B*, **33**, 8822-8824 (1986).

7. Grimme, S. Ehrlich, S. Goerigk, L. Effect of the damping function in dispersion corrected density functional theory. *J. Comput. Chem*. **32**, 1456-1465 (2011).

8. Grimme, S. Antony, J. Ehrlich, S. Krieg, H. A consistent and accurate ab initio parametrization of density functional dispersion correction (DFT-D) for the 94

elements H-Pu. *J. Chem. Phys*. **132**, 154104 (2010).

9. Schäfer, A. Horn, H. Ahlrichs, R. Fully optimized contracted Gaussian basis sets for atoms Li to Kr. *J. Chem. Phys.* **97**, 2571-2577 (1992).

10. Weigend, F.; Ahlrichs, R. Balanced basis sets of split valence, triple zeta valence and quadruple zeta valence quality for H to Rn: Design and assessment of accuracy. *Phys. Chem. Chem. Phys*. **7**, 3297-3305 (2005).

11. Weigend, F. Accurate Coulomb-fitting basis sets for H to Rn. *Phys. Chem. Chem. Phys*. **8**, 1057-1065 (2006).

12. Dolg, M. Stoll, H. Preuss, H. Pitzer, R.M. Relativistic and correlation effects for element 105 (hahnium, Ha): a comparative study of M and MO (M= Nb, Ta, Ha) using energy-adjusted ab initio pseudopotentials. *J. Phys. Chem.* **97**, 5852-5859 (1993).

13. Glendening, E. D.; Landis, C. R.; Weinhold, F. NBO 6.0: Natural bond orbital analysis program. *J. Comput. Chem*. **34**, 1429-1437 (2013).

14. Frisch, M. J.; Trucks, G. W.; Schlegel, H. B.; Scuseria, G. E.;Robb, M. A.; Cheeseman, J. R.; Montgomery, J. A., Jr.; Vreven, T.; Kudin, K. N.; Burant, J. C.; Millam, J. M.; Iyengar, S. S.; Tomasi, J.; Barone, V.; Mennucci, B.; Cossi, M.; Scalmani, G.; Rega, N.; Petersson, G. A.; Nakatsuji, H.; Hada, M.; Ehara, M.; Toyota, K.; Fukuda, R.; Hasegawa, J.; Ishida, M.; Nakajima, T.; Honda, Y.; Kitao, O.; Nakai, H.; Klene, M.; Li, X.; Knox, J. E.; Hratchian, H. P.; Cross, J. B.; Bakken, V.; Adamo, C.; Jaramillo, J.; Gomperts, R.; Stratmann, R. E.; Yazyev, O.; Austin, A. J.; Cammi, R.; Pomelli, C.; Ochterski, J. W.; Ayala, P. Y.; Morokuma, K.; Voth, G. A.; Salvador, P.;

Dannenberg, J. J.; Zakrzewski, V. G.; Dapprich, S.; Daniels, A. D.; Strain, M. C.; Farkas, O.; Malick, D. K.; Rabuck, A. D.; Raghavachari, K.; Foresman, J. B.; Ortiz, J. V.; Cui, Q.; Baboul, A. G.; Clifford, S.; Cioslowski, J.; Stefanov, B. B.; Liu, G.; Liashenko, A.; Piskorz, P.; Komaromi, I.; Martin, R. L.; Fox, D. J.; Keith, T.; Al-Laham, M. A.; Peng, C. Y.; Nanayakkara, A.; Challacombe, M.; Gill, P. M. W.; Johnson, B.; Chen, W.; Wong, M. W.; Gonzalez, C.; Pople, J. A. Gaussian 09, revision D.01; Gaussian, Inc., Wallingford, CT, **2013**.

15. Bader, R, F. W. Atoms in Molecules: A Quantum Theory. USA: Oxford University Press, **1994**.

16. AIMAll (Version 17.11.14), Keith, T. A. TK Gristmill Software, Overland Park KS, USA, **2017** (aim.tkgristmill.com).

17. Ziegler, T.; Rauk, A. On the calculation of bonding energies by the Hartree Fock Slater method. *Theor. Chim. Acta*, **46**, 1-10 (1977).

18. Mitoraj, M.; Michalak, A. Donor–acceptor properties of ligands from the natural orbitals for chemical valence. *Organometallics*, **26**, 6576-6580 (2007).

19. Mitoraj, M.; Michalak, A. Applications of natural orbitals for chemical valence in a description of bonding in conjugated molecules. *J. Mol. Model.* **14**, 681-687 (2008).

20. ADF2017, SCM, Theoretical Chemistry, Vrije Universiteit, Amsterdam, The Netherlands, http://www.scm.com

21. Bickelhaupt, te Velde, G. Baerends, F. M. Guerra, E. J. Van Gisbergen, C. F. Snijders, S. J. A. Ziegler, T. Chemistry with ADF. *J. Comput. Chem.* **22**, 931-967 (2001).

22. Michalak, A. Mitoraj, M. Ziegler, T. Bond orbitals from chemical valence theory. *J. Phys. Chem. A*, **112**, 1933-1939 (2008).

23. Mitoraj, M. P.; Michalak, A.; Ziegler, T. A combined charge and energy decomposition scheme for bond analysis. *J. Chem. Theory Comput.* **5**, 962-975 (2009).

24. van Lenthe, E., Baerends, E. J. Optimized Slater-type basis sets for the elements 1-118. *J. Comput. Chem*. **24**, 1142-1156 (2003).

25. Zhao, L. von Hopffgarten, M. Andrada, D. M. Frenking, G. Energy decomposition analysis. *WIREs Comput. Mol. Sci.,* **8**, 1345 (2018).

26. Frenking, G. Bickelhaupt, F. M. The EDA Perspective of Chemical Bonding. In *The Chemical Bond 1. Fundamental Aspects of Chemical Bonding*, G. Frenking, S. Shaik, Eds. Wiley-VCH: Weinheim, 121-158 (2014).
