# Supplementary material for: Double dative bond between divalent carbon(0) and uranium
Source: Nat Commun. 2018 Nov 27;9:4997. doi: 10.1038/s41467-018-07377-6 (PMC6258733; doi:10.1038/s41467-018-07377-6)
Supplement: Supplementary file 2 — Supplementary Data [file 41467_2018_7377_MOESM2_ESM.pdf]

Coordinates and energies of the calculated molecules at the BP86-D3(BJ)/def2-TZVPP/Stuttgart RSC ECP level.

Complex 2

E = -4462.11139393 au

0 3

U	-2.18064400	-0.13766100	-0.02340900
Cl	-2.24694900	-2.46927900	1.15294100
Cl	-4.74526000	0.09091900	-0.12725000
Cl	-2.20354200	0.74634500	-2.53542600
Cl	-2.01068600	0.73438300	2.47452700
P	0.97518400	1.50723400	0.24871800
P	1.09442900	-1.40253000	-0.25709900
C	-1.55355400	-3.79723400	-3.09055100
H	-2.24440200	-4.30444700	-3.76299900
C	-0.31809800	2.79321500	0.28457500
C	-0.01098000	4.13932300	0.48687700
H	1.01800000	4.43977800	0.68588800
C	-1.04238600	5.07660900	0.42116900
H	-0.83919700	6.13527900	0.58400100
C	-2.55787900	3.27253500	-0.06729500
H	-3.54831300	2.87396300	-0.29413300
C	-2.33602900	4.63323500	0.13144900
H	-3.16949900	5.33106900	0.06007200
C	2.71924000	-1.27748300	-1.06959200
C	3.92399400	-1.42188000	-0.36636000
H	3.90743000	-1.75062500	0.67268600
C	2.74299700	-0.85931200	-2.41113000
H	1.80476200	-0.73313500	-2.95268400
C	3.95699100	-0.58016000	-3.03536100
H	3.96688000	-0.24233500	-4.07161000
C	5.13910000	-1.13984300	-0.99651800
H	6.07352200	-1.25173600	-0.44490000
C	5.15624800	-0.71431700	-2.32731500
H	6.10480200	-0.48635900	-2.81500700
C	1.76321500	-3.57422300	3.74690900
H	1.91303700	-4.07677800	4.70360400
C	0.23688700	0.03727500	-0.09392900
C	1.37288800	-2.29576100	1.29725200
C	0.09763900	-2.43857200	-1.38243700
C	0.59892500	-3.55777800	-2.04859500

H	1.63536900	-3.86221300	-1.90455200
C	3.70793200	0.83333600	3.20535300
H	4.68970100	0.36594800	3.29150400
C	2.05626100	-3.52088800	1.34560700
H	2.43934100	-3.97754600	0.43159300
C	0.87159600	-1.71979400	2.47254000
H	0.29829700	-0.79317900	2.42074400
C	1.86110000	1.59494300	1.82731900
C	1.45566000	2.07534200	-2.37113000
H	0.43818300	1.68751200	-2.48591900
C	2.24931800	-4.15861200	2.57118600
H	2.77552800	-5.11327600	2.61013700
C	2.04871500	2.10977100	-1.09418900
C	-0.24500200	-4.25480900	-2.91464400
H	0.11516800	-5.13372200	-3.44990500
C	2.17294400	2.52514500	-3.47746000
H	1.70788600	2.50766200	-4.46395100
C	3.12867800	0.99861900	1.94829900
H	3.64975200	0.63734200	1.06200700
C	-1.97411400	-2.66733000	-2.39144600
H	-2.98345600	-2.26785500	-2.50290300
C	1.17551800	2.01061400	2.98086300
H	0.16821900	2.41835300	2.90086400
C	4.07321300	3.02018800	-2.05756400
H	5.09500900	3.38219700	-1.93648800
C	3.35125700	2.59683900	-0.93677200
H	3.80339400	2.64917600	0.05303000
C	3.48603700	2.98702100	-3.32423300
H	4.05024400	3.32573300	-4.19439400
C	1.07507700	-2.35926700	3.69546200
H	0.67578100	-1.91067100	4.60514900
C	3.02375400	1.24991000	4.35212100
H	3.47209100	1.10793800	5.33621300
C	1.76027600	1.83582800	4.23663200
H	1.21527900	2.14575000	5.12882200
N	-1.16877300	-2.00272300	-1.54583400
N	-1.56734000	2.36491000	0.02133100

Complex 4

E = -4446.05867637 au

0 3

U	2.30197800	0.18621300	0.02029000
Cl	4.84531900	-0.18034800	0.04571200
Cl	2.17464600	2.70582800	0.49106300

Cl	2.21733800	-0.46591200	2.55126300
Cl	2.35925700	-0.24059500	-2.56112000
P	-1.20728300	1.38570800	-0.13119300
P	-0.83666800	-1.49516500	0.08323900
C	2.56537800	-4.53764600	0.11643500
H	3.41464000	-5.21871300	0.07826200
C	-0.58086700	2.68130000	-1.23569800
C	0.04393900	2.28083700	-2.42557600
H	0.19891500	1.22191500	-2.62986100
C	-0.70483900	4.04415700	-0.93580600
H	-1.14413200	4.36279700	0.00807500
C	0.54517900	3.23829000	-3.30756900
H	1.05941800	2.91443300	-4.21275400
C	-0.20593700	4.99681700	-1.82412200
H	-0.28356000	6.05617300	-1.57678700
C	0.41859100	4.59716600	-3.00982000
H	0.82430000	5.34611400	-3.69117600
C	-1.79659800	-1.78019200	1.60252500
C	-3.19734200	-1.74585400	1.66954100
H	-3.78934100	-1.65156600	0.76301600
C	-1.04837100	-1.85527600	2.79317800
H	0.04299800	-1.83780900	2.75537600
C	-1.69797000	-1.89846800	4.02522100
H	-1.10721000	-1.94506600	4.94058400
C	-3.84132900	-1.79322400	2.90840400
H	-4.93111500	-1.76395800	2.94965700
C	-3.09543400	-1.86882600	4.08696600
H	-3.60120900	-1.89885100	5.05287600
C	-2.90096900	-2.94666900	-3.78619500
H	-3.35940300	-3.26820200	-4.72236800
C	-0.15130800	0.04016400	-0.10386800
C	-1.74569300	-2.10019400	-1.38096500
C	0.50204400	-2.73643400	0.18165700
C	0.21962100	-4.08733800	0.38633400
H	-0.80751900	-4.40723500	0.56130000
C	-5.20605900	0.40479700	-0.46504900
H	-6.05483700	0.25459600	0.20388700
C	-2.87552100	-2.92875900	-1.36303200
H	-3.30235300	-3.26448700	-0.41963700
C	-1.17096700	-1.72938400	-2.61055600
H	-0.26884000	-1.11212900	-2.61612600
C	-2.87649700	1.00912700	-0.78625800
C	-0.72643700	1.66941600	2.57957000
H	0.04352700	0.91495400	2.41579600

C	-3.46011400	-3.33452400	-2.56624000
H	-4.35321000	-3.96048700	-2.54736200
C	-1.50559600	2.11543200	1.50526100
C	1.27007800	-5.00515700	0.35955100
H	1.08139500	-6.06664900	0.52101500
C	-0.92928400	2.20058200	3.85455600
H	-0.31141000	1.85140300	4.68224800
C	-3.97535000	0.80744200	0.06116500
H	-3.86704300	0.95458400	1.13457700
C	2.77014700	-3.17357500	-0.06725600
H	3.76248100	-2.75166100	-0.23464500
C	-3.03368500	0.81757000	-2.16941100
H	-2.19219600	0.99139000	-2.83877500
C	-2.69175200	3.62092800	2.99117700
H	-3.45897100	4.37967300	3.15109900
C	-2.49433800	3.09349900	1.71485700
H	-3.11802500	3.43145200	0.88614800
C	-1.90848900	3.17469500	4.06223400
H	-2.06480900	3.58972500	5.05903800
C	-1.74472500	-2.15888300	-3.80551300
H	-1.28869500	-1.87350600	-4.75423500
C	-5.34658400	0.18969400	-1.83768700
H	-6.30480900	-0.13418300	-2.24585100
C	-4.25721800	0.40091400	-2.68953200
H	-4.35976500	0.23916400	-3.76273600
N	1.75461600	-2.28295900	-0.02663400

Complex 6
E = -4429.99935800 au

0 3

U	2.21021900	0.28248100	-0.05902900
Cl	2.44086900	2.81490100	0.00036700
Cl	4.59310100	-0.62893100	0.06824600
Cl	2.24517600	-0.13283600	-2.62888300
Cl	2.10286500	0.15244300	2.54403400
P	-0.65969100	-1.54961800	0.11465900
P	-1.36459500	1.33435100	-0.15178200
C	0.18383700	4.83180800	-2.70022700
H	0.57008200	5.65648700	-3.30066400
C	0.83999300	-2.56998700	0.30526500
C	1.33857800	-2.92962000	1.57010000
H	0.81360900	-2.62002100	2.47171600
C	1.53510200	-2.96324600	-0.85891200

H	1.15379400	-2.68779000	-1.84138300
C	2.51954500	-3.66688200	1.66696100
H	2.91037500	-3.92718200	2.65038500
C	2.72298800	-3.68772900	-0.74791400
H	3.27282200	-3.95717800	-1.64905600
C	3.21271800	-4.03981400	0.51248500
H	4.14887300	-4.59222500	0.59565700
C	-2.97572600	0.89336200	-0.89368500
C	-3.81354500	-0.03483700	-0.25256700
H	-3.57113900	-0.38921000	0.74703300
C	-3.32047900	1.35634500	-2.17286700
H	-2.68513600	2.08566000	-2.67481400
C	-4.46336700	0.87007900	-2.81146400
H	-4.71753200	1.23174500	-3.80863700
C	-4.95216200	-0.51954300	-0.89450500
H	-5.58580900	-1.24876800	-0.38817100
C	-5.27441500	-0.07699600	-2.18078100
H	-6.15930200	-0.46417200	-2.68722300
C	-2.15172900	2.87788400	4.12782200
H	-2.31514400	3.20883000	5.15451000
C	-0.21430700	0.06875500	-0.14702500
C	-1.73298300	1.99865700	1.50334700
C	-0.75862000	2.72462400	-1.13329600
C	-0.98126600	4.04693000	-0.72786900
H	-1.49904300	4.25169800	0.20919400
C	-3.57273400	-2.62904300	2.86005400
H	-4.47127700	-3.24618800	2.90259700
C	-0.07663800	2.45676600	-2.32971200
H	0.13252900	1.42685800	-2.62124800
C	-3.03773200	2.16614200	1.98895600
H	-3.89300800	1.95693100	1.34801200
C	-0.63761700	2.30531900	2.32817300
H	0.38087700	2.18701700	1.95821000
C	-1.66036300	-1.79829700	1.62263200
C	-1.70827200	-1.62815500	-2.45864300
H	-1.36031400	-0.59796000	-2.50568700
C	-3.24340300	2.59767800	3.30206400
H	-4.26051300	2.71391400	3.67859000
C	-1.50303000	-2.35895300	-1.28372000
C	-0.50419500	5.09801900	-1.51231800
H	-0.65592400	6.12765700	-1.18660100
C	-2.32666200	-2.22691600	-3.55723500
H	-2.48873500	-1.64819400	-4.46689300
C	-2.80800300	-2.60295400	1.69162800

H	-3.13067900	-3.17623300	0.82379500
C	0.39352300	3.51210400	-3.11003500
H	0.94944400	3.29775400	-4.02290800
C	-1.28365900	-1.02801700	2.73761500
H	-0.39655500	-0.39532600	2.68220400
C	-2.47701300	-4.31344000	-2.33542200
H	-2.75540500	-5.36729600	-2.29543900
C	-1.85983000	-3.71698400	-1.23563700
H	-1.63137900	-4.31399700	-0.35159700
C	-2.72358100	-3.56479000	-3.49215600
H	-3.20653400	-4.03377900	-4.35043400
C	-0.84908300	2.74256100	3.63428000
H	0.00965100	2.96756800	4.26730200
C	-3.19775600	-1.85783100	3.96500500
H	-3.80433300	-1.87351500	4.87148800
C	-2.05002900	-1.06236100	3.90269800
H	-1.75283000	-0.45101600	4.75500500
