# Supplementary material for: Double dative bond between divalent carbon(0) and uranium
Source: Nat Commun. 2018 Nov 27;9:4997. doi: 10.1038/s41467-018-07377-6 (PMC6258733; doi:10.1038/s41467-018-07377-6)
Supplement: Supplementary file 3 — Description of Additional Supplementary Files [file 41467_2018_7377_MOESM3_ESM.docx]

Description of Additional Supplementary Files

Supplementary Dataset: Coordinates and energies of the calculated molecules.
